# Supplementary material for: Two-layer homolog network approach for PFAS nontarget screening and retrospective data mining
Source: Nat Commun. 2025 Jan 15;16:688. doi: 10.1038/s41467-025-56035-1 (PMC11735632; doi:10.1038/s41467-025-56035-1)
Supplement: Supplementary file 1 — Supplementary Information [file 41467_2025_56035_MOESM1_ESM.pdf]

# Two-layer homolog network approach for PFAS nontarget screening and retrospective data mining

Zhaoyu Jiao[1], Sachi Taniyasu[2], Nanyang Yu[1], Xuebing Wang[1], Nobuyoshi Yamashita[2], and Si Wei[1*]

[1]State Key Laboratory of Pollution Control and Resource Reuse, School of the Environment, Nanjing University, Nanjing, People's Republic of China

[2]National Institute of Advanced Industrial Science and Technology (AIST), 16-1 Onogawa, Tsukuba, Ibaraki 305-8569, Japan

[*] E-mail: weisi@nju.edu.cn

**Contents**

**Supplementary Notes..........................................................................Page S3**

**Supplementary Methods. .....................................................................Page S4**

**Supplementary Tables... ......................................................................Page S8**

**Supplementary Figures....................................................................Page S11**

**Supplementary Notes**

**Supplementary Note 1. The identification of PFAS**

The unsaturated fragment of 504.97101 Da in the spectra of Node 235 was identified as $[C_{12}F_{19}]^-$ similar to other nodes in the community a (Supplementary Fig. 16). The predominant fragment of 87.00756 Da corresponds to $[C_3H_3O_3]^-$. The precursor of 606.98328 Da only matched $C_{13}H_4F_{21}O_3$ within 5 ppm tolerance after setting the minimum element composition to $C_{12}H_3F_{19}O_3$. This class was finally identified as n:3 FTECAs, which could reasonably explain the presence of $[C_{12}F_{19}]^-$ and $[C_3H_3O_3]^-$.

A series of saturated fluorocarbon fragments were observed in the spectra of community c (Supplementary Fig. 23), where PFCAs are present. Fragment of 85.02827 Da corresponding to $[C_4H_5O_2]^-$ was observed in the spectra of Node 18. Another group of spectra was also found in Node 18. The fragment of 62.98739 Da was identified as $[FCO_2]^-$, indicating the presence of the carboxyl group. Three adjacent fragments were identified as $[C_9F_7]^-$, $[C_9HF_8]^-$ and $[C_9H_2F_9]^-$, between which the neutral loss was HF. The neutral loss of 124.0169 Da was identified as $H_4F_4CO_2$, which is similar to n:3 FTCAs and n:2 FTOHs. Therefore, Node 18 was finally identified as n:4 FTCAs and branched isomeric n:4 FTCAs. Other nodes in this community were found to be adducts and fluorocarbon fragments of PFCAs, H-PFCAs and n:4 FTCAs (Supplementary Figure 19 and 23).

Unsaturated fragments of $[C_nF_{2n-3}]^-$, $[C_nF_{2n-1}]^-$ were observed in the spectra of community d (Supplementary Fig. 24). The neutral loss of 20.00915 Da of Node 404 corresponds to HF, leading to the identification of $[C_6HF_{10}]^-$ (Supplementary Fig. 20). The terminal neutral loss of 43.98763 Da correspond to $CO_2$, indicating the presence of the carboxyl group. Node 404 was finally identified as dH-PFCAs. However, position of the double bond and the hydrogen were still uncertain considering that we observed both $[C_3F_3]^-$ and $[C_2F_5]^-$ in the spectral of this class. One of the C7-isomers which have similar spectrum with this class (Supplementary Fig. 20c) have been recorded. A series of unsaturated carbon fluorine fragments including $[C_3F_5]^-$, $[C_4F_7]^-$, $[C_6F_7]^-$ and $[C_6F_9]^-$ were observed in the spectra of node 166 (Supplementary Fig. 21). The neutral loss of

27.99874 Da corresponds to CO, contributing to the identification of [$C_7F_9O$]-. Subsequently, a series of neutral loss of HF were observed, leading to the diagnosis of [$C_7HF_{10}O$]-, [$C_7H_2F_{11}O$]- and [$C_7H_3F_{12}O$]-. The fragment pattern is consistent with fluoropolymer. Finally, this class was identified as H-n:1 FTOHs. The predominant fragment of node 422 (Supplementary Fig. 22) was identified as [$C_7F_{13}$]-. The neutral loss of 27.99832 Da corresponds to CO and the adjacent fragment was identified as [$C_8F_{13}O$]-, indicating the presence of the ether bond. The terminal neutral loss corresponds to $H_2F_2CO_2$, indicating the presence of the carboxyl group. Therefore, this class was identified as n:2 FTECAs.

**Supplementary Note 2. Evaluation of internal network**

We compared our screening results with FluoroMatch 4.5 and FindPFΔS using the standard sample. We found that our approach has fewer false positive and false negative features (Supplementary Fig. 1c) than FluoroMatch. This is reasonable considering that FluoroMatch is based on the homolog screening. Our approach demonstrates comparable false positives and significantly lower false negatives relative to FindPFΔS, which employs MS/MS spectra information for prioritization. This evaluation suggests that our approach effectively integrates MS1 and MS/MS information, thereby leveraging the strengths of current strategies. However, there are certain limitations in this comparison. Specifically, the parameters for FluoroMatch and FindPFΔS were not further optimized to suit the dataset, and the evaluation was based on a limited standard sample. The total number of non-PFAS features in the standard samples is difficult to quantify due to differences in peak detection methods across approaches. Therefore, we calculated the number of false positive features and false negative features instead of the FPR and FNR.

**Supplementary Note 3. Parameter setting of MASST**

The parameters of MS tolerance, matched peaks and cosine scores were chosen based on the following reasons.

Mass Accuracy Settings: We tested the mass accuracy using PFAS standards. Results (shown in Supplementary Fig. 55) indicate that the precursor mass errors were within

±0.002 Da. Considering the typical mass error of high-resolution mass spectrometers in MassIVE and settings used in other MassIVE studies (e.g., doi.org/10.1038/s41586-023-06906-8, doi.org/10.1038/s41467-022-34537-6), we set the precursor mass error to 0.005 Da and the MS/MS fragment ion error to 0.01 Da.

Minimum Matched Fragments: PFAS compounds typically generate fewer fragments compared to metabolites. Statistical analysis of the MS/MS spectra from 94 identified PFAS compounds (Supplementary Fig. 56) revealed that 13% of the PFAS compounds had only two effective fragments. Additionally, we assessed the discovery rate and false discovery rate using samples at concentrations of 100 μg/L and 1 μg/L by searching an in-house PFAS dataset (see Supplementary methods). Our results demonstrated that increasing the minimum number of matched fragments from 2 to 3 led to a 20% reduction in discovery rate at 100 μg/L and a 29% reduction at 1 μg/L (Supplementary Fig. 57 b&d). Moreover, this parameter setting aligns with the approach used in two MassIVE-related studies.

Cosine Score Threshold: We tested the discovery rate at different similarity thresholds using reference standards and found a significant decrease in the discovery rate when the threshold was set above 0.6 (Supplementary Fig.57 d). To balance the discovery rate and false discovery rate, we ultimately decided to set the threshold at 0.6. We evaluated the false discovery rate by searching 35 PFAS standards and 830 local PFAS spectra against 16,734 non-PFAS and 2,207 blank features. The false discovery rate was 0 and 0.017 (Supplementary Fig. 57) under a similarity threshold of 0.6 and 2 matched fragments, which is considered acceptable.

**Supplementary Methods**

**Evaluation of internal network**

We evaluated the denoising capability of the internal network using a local mass spectra database and an in-house standard sample (100 μg/L). The local spectra database contains 133 PFAS from 45 classes, while the standard samples include 32 PFAS from 6 classes. MS1 tolerance was set at 5 ppm for homolog screening and internal network (Supplementary Fig. 1a). The parameters for FluoroMatch were set to:

Full-scan Intensity threshold: 10000, m/z Search Tolerance MS1: 0.002, Scan filter in seconds: 0-3600, m/z Search Window MS/MS: 10 ppm, MS/MS intensity threshold (file conversion): 500, MS/MS intensity threshold (annotation): 500, Blank Filtering: 3. These parameters are similar to or consistent with those used in our approach. We utilized the "fragment differences" module of FindPFΔS for comparative analysis. The fragment difference settings were configured as follows: FindPFAS_1: $CF_2$, FindPFAS_2: $CF_2$, $C_2F_4$, HF, and FindPFAS_3: $CF_2$, $C_2F_4$, HF, $CF_3$, $CF_2O$. The parameters for these configurations were set to: Number of Differences Desired: 1.0, Fragment Mass Tolerance: 0.01 Da, Intensity Threshold: 5.0, Remove Multiple Mass Tolerance: 0.002, and Occurrence Number Threshold: 20.0. These parameters are consistent with our settings or are inconsequential to the results. Additionally, we evaluated the analytical method using typical fragments with the “diagnostic fragment” module of FindPFΔS. For this evaluation, the fragments were set to the default values, including $[CF_3]$-, $[PO_2F]$-, $[SO_2F]$-, $[C_2F_5]$-, $[C_3F_7]$-, $[C_4F_9]$-, $[C_5F_{11}]$-, $[C_6F_{13}]$-, and $[C_7F_{15}]$-.

False positive features refer to non-PFAS that were incorrectly identified as PFAS, and false negative features refer to PFAS that were not identified as PFAS. The false positive rate (FPR) was calculated by dividing the number of false positive features by the number of non-PFAS in the database. The false negative rate (FNR) was calculated by dividing the number of false negative features by the number of PFAS in the database. The total number of non-PFAS features in the standard samples is difficult to quantify due to varying peak detection methods across approaches. Therefore, the FPR and FNR were not calculated for the standard sample.

**Validation of MASST search settings**

We evaluated the MASST search performance using PFAS standards (35 PFAS), a menthol blank sample (2207 spectra), and a local spectra database (830 PFAS and 16,734 non-PFAS). We optimized MASST search parameters in two key areas:

1) Mass Accuracy Settings: This includes the precursor mass error and MS/MS fragment error.

2) Spectra Match Settings: This encompasses the minimum number of matched fragments and cosine score thresholds.

Detailed Evaluations:

1) Mass Accuracy Settings: We tested the mass accuracy using 35 PFAS standards across various concentrations (0.5, 1, 2, 5, 10, 20, 50, 100 μg/L).

2) Minimum Matched Fragments and Cosine Score Threshold: We assessed MASST search performance under different parameter settings, using both 100 μg/L and 1 μg/L samples to evaluate discovery rates (against 830 local PFAS spectra) and false discovery rates (against 16,734 local non-PFAS spectra and 2207 blank sample spectra). Discovery rate and false discovery rate were defined as follows:

Discovery Rate = Number of true PFAS / Number of used PFAS

False Discovery Rate = Number of false non-PFAS / Number of used non-PFAS

**Supplementary Table 1. Diagnostic fragments and neutral losses of identified classes.**

| ID | Class | Chain length (n) | Diagnostic fragments | Diagnostic neutral losses |
|---|---|---|---|---|
| 1 | PFCA | n=2-14 | $C_2F_5$-, $C_3F_7$- | |
| 2 | PFSA | n=8 | $SO_3$-, $SO_3F$- | |
| 3 | H-PFCA | n=4-17 | $C_2HF_4$-, $C_2F_5$- | |
| 4 | n:3 FTCA | n=8-15 | $CFO_2$-, $C_7F_7$-, $C_{n-2}F_{2n-11}$-, $C_{n-1}F_{2n-9}$-, $C_{n-1}HF_{2n-8}$- | HF, $H_3F_3CO_2$ |
| 5 | PA | n=2-4 | $C_2F_5$-, $C_3F_7$- | $CF_2O$ |
| 6 | Cl-PFCA[a] | n=9, 11-14 | | |
| 7 | n:2 FTOH | n=10, 12, 14, 16 | $C_2F_5$-, $C_{n-3}F_{2n-13}$-, $C_{n-1}F_{2n-9}$-, $C_{n-1}F_{2n-7}$-, $C_nF_{2n-7}O$-, $C_nHF_{2n-6}O$- | CO, HF, $H_4F_4CO$ |
| 8 | n:2 FTSF | n=8, 10, 12, 14, 16 | $SO_4H$- | |
| 9 | BPAF | n=15, 18 | $CF_3$-, $C_{14}H_8F_3O_2$- | $CHF_3$ |
| 10 | PFTrECA | n=11-12 | $C_2F_5O$-, $C_5F_{11}O_2$-, $C_8F_{17}O_3$- | $C_3F_6O$, $C_2F_4CO_2$ |
| 11 | PFTeECA | n=14-15 | $C_2F_5O$-, $C_5F_{11}O_2$-, $C_8F_{17}O_3$-, $C_{11}F_{23}O_4$- | $C_3F_6O$, $C_2F_4CO_2$ |
| 12 | PFPeECA | n=17-18 | $C_2F_5O$-, $C_5F_{11}O_2$-, $C_8F_{17}O_3$-, $C_{11}F_{23}O_4$-, $C_{14}F_{29}O_5$- | $C_3F_6O$, $C_2F_4CO_2$ |
| 13 | n:2:3 FTECA | n=11-17, 19 | $C_3H_5O_3$-, $C_{n-4}F_{2n-15}$-, $C_{n-4}F_{2n-13}$-, $C_{n-3}F_{2n-13}O$-, $C_{n-3}HF_{2n-12}O$-, $C_{n-3}H_2F_{2n-11}O$- | CO, HF |
| 14 | n:3 FTECA | n=8-11, 13 | $C_3H_3O_3$-, $C_{n-1}F_{2n-7}$- | |
| 15 | n:4 FTCA | n=10, 12, 14 | $FCO_2$-, $C_{n-1}F_{2n-13}$-, $C_{n-1}HF_{2n-12}$-, $C_{n-1}H_2F_{2n-11}$- | HF, $H_4F_4CO_2$ |
| 15_2 | m-n:4 FTCA | n=12, 14, 16 | $C_4H_5O_2$-, $C_2F_5$-, $C_5F_9$-, $C_8F_{15}$- | |
| 16 | dH-PFCA | n=7-10, 12 | $C_2F_5$-, $C_{n-1}F_{2n-5}$, $C_{n-1}HF_{2n-4}$ | $CO_2$ |
| 17 | H-n:1 FTOH | n=7, 9, 11, 13, 15 | $C_3F_5$-, $C_4F_7$-, $C_{n-1}F_{2n-7}$-, $C_nF_{2n-5}O$-, $C_nHF_{2n-4}O$-, $C_nH_2F_{2n-3}O$- | CO, HF |
| 18 | n:2 FTECA | n=7, 9 | $C_{n-2}F_{2n-7}$-, $C_{n-2}F_{2n-5}$-, $C_{n-1}F_{2n-5}O$- | $H_2F_2CO_2$ |

Note: [a] a distinct isotope pattern M:M+2=3:1 in this class.

**Supplementary Table 2. Peak areas of identified PFAS class.**

| Class | Rubber car wiper | Waterproof cloth | Textile | Waterproof chemical | Industrial sludge |
|---|---|---|---|---|---|
| PFCAs | 1.31E+07 | 1.28E+07 | 1.27E+07 | 8.15E+08 | 8.34E+08 |
| PFSAs | 2.06E+06 | 4.58E+06 | 5.41E+06 | 3.41E+06 | 4.99E+06 |
| H-PFCAs | 2.58E+06 | 2.46E+06 | 2.09E+06 | 2.43E+07 | 7.71E+08 |
| n:3 FTCAs | 7.26E+05 | 0.00E+00 | 5.44E+05 | 1.65E+08 | 7.38E+06 |
| Cl-PFCAs | 0.00E+00 | 0.00E+00 | 0.00E+00 | 0.00E+00 | 4.83E+07 |
| PAs | 0.00E+00 | 0.00E+00 | 0.00E+00 | 6.76E+04 | 2.32E+07 |
| n:2 FTOHs | 0.00E+00 | 0.00E+00 | 0.00E+00 | 8.21E+07 | 0.00E+00 |
| BPAFs | 0.00E+00 | 0.00E+00 | 0.00E+00 | 0.00E+00 | 3.39E+09 |
| n:4 FTCAs | 0.00E+00 | 0.00E+00 | 0.00E+00 | 8.87E+07 | 0.00E+00 |
| PFTrECAs | 0.00E+00 | 0.00E+00 | 0.00E+00 | 0.00E+00 | 4.41E+07 |
| PFTeECAs | 0.00E+00 | 0.00E+00 | 0.00E+00 | 0.00E+00 | 1.39E+08 |
| PFPeECAs | 0.00E+00 | 0.00E+00 | 0.00E+00 | 0.00E+00 | 1.05E+07 |
| dH-PFCAs | 0.00E+00 | 0.00E+00 | 0.00E+00 | 2.59E+08 | 0.00E+00 |
| H-n:1FTOH | 1.29E+06 | 0.00E+00 | 1.60E+06 | 0.00E+00 | 7.29E+07 |
| n:2 FTECAs | 0.00E+00 | 0.00E+00 | 0.00E+00 | 1.14E+07 | 0.00E+00 |
| n:3 FTECAs | 2.95E+06 | 0.00E+00 | 1.94E+06 | 4.37E+07 | 1.25E+06 |
| n:2:3 FTECAs | 7.03E+06 | 0.00E+00 | 2.23E+06 | 6.81E+09 | 1.12E+07 |
| FTSFs | 1.14E+06 | 1.33E+05 | 0.00E+00 | 9.02E+08 | 9.94E+05 |

**Supplementary Table 3. Mobile phase gradient elution condition.**

| Time | Flow rate (μL/min) | A(%) | B(%) |
|---|---|---|---|
| 0 | 250 | 90 | 10 |
| 1 | 250 | 90 | 10 |
| 36 | 250 | 0 | 100 |
| 50 | 250 | 0 | 100 |
| 50.1 | 250 | 90 | 10 |
| 55 | 250 | 90 | 10 |

**Supplementary Table 4. Recoveries and relative standard deviations (RSD) of PFAS in the instrument concentration of 20 μg/L.**

| PFAS | Formula | Recovery | RSD |
|---|---|---|---|
| PFBA | C4HF7O2 | 91.1% | 5.0% |
| PFPeA | C5HF9O2 | 93.2% | 8.4% |
| PFHxA | C6HF11O2 | 79.0% | 12.1% |
| PFHpA | C7HF13O2 | 97.0% | 8.8% |
| PFOA | C8HF15O2 | 93.5% | 8.1% |
| PFNA | C9HF17O2 | 86.3% | 5.0% |
| PFDA | C10HF19O2 | 88.4% | 10.5% |
| PFUdA | C11HF21O2 | 86.3% | 7.2% |
| PFDoA | C12HF23O2 | 91.1% | 1.4% |
| PFTrDA | C13HF25O2 | 99.7% | 12.7% |
| PFTeDA | C14HF27O2 | 89.2% | 12.7% |
| PFHxDA | C16HF31O2 | 74.3% | 14.4% |
| PFODA | C18HF35O2 | 76.2% | 6.9% |
| PFBS | C4HF9O3S | 93.1% | 14.2% |
| PFPeS | C5HF11O3S | 107.7% | 6.2% |
| PFHxS | C6HF13O3S | 98.7% | 8.8% |
| PFHpS | C7HF15O3S | 91.0% | 8.3% |
| PFOS | C8HF17O3S | 87.0% | 4.6% |
| PFNS | C9HF19O3S | 91.2% | 10.7% |
| PFDS | C10HF21O3S | 84.5% | 7.9% |
| PFDoS | C12HF25O3S | 96.7% | 10.7% |

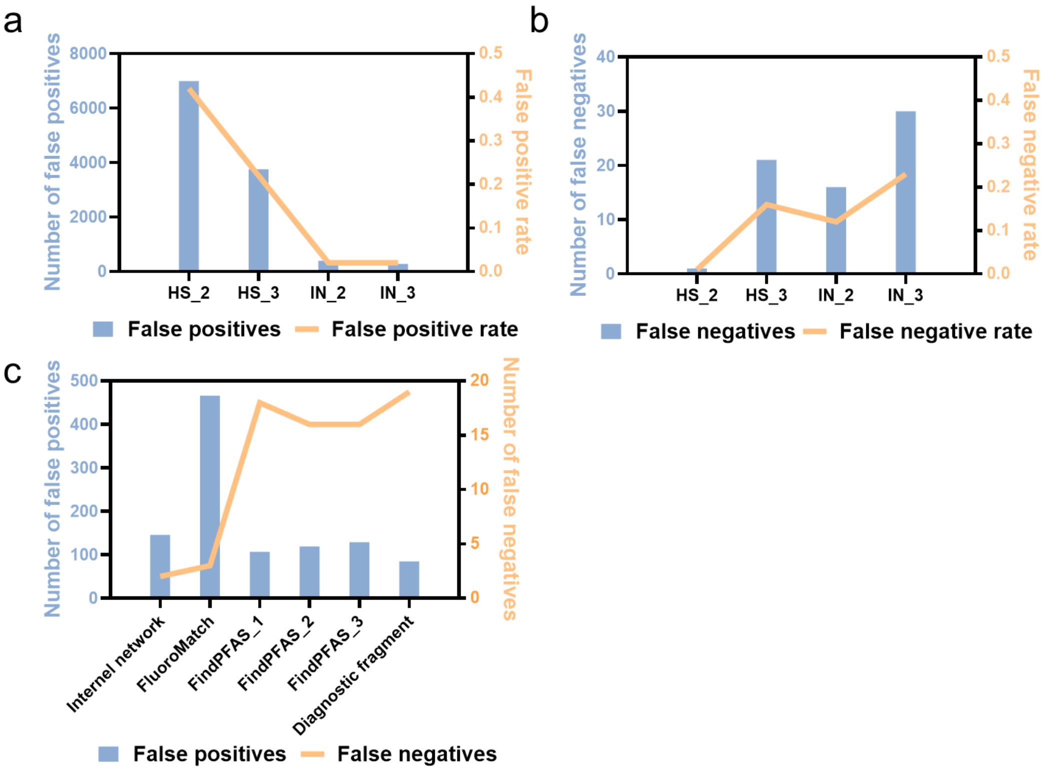


**Supplementary Figure 1. The comparison of homolog network with other methods. a** The number of false positive features and false positive rates of homolog screening (HS) and internal network (IN) using the local mass spectrometry database. HS_2 and HS_3 refers to HS with a minimum of 2 homologs per class and 3 homologs per class, respectively. IN_2 and IN_3 refers to IN with a minimum of 2 homologs per class and 3 homologs per class, respectively. **b** The number of false negative features and false negative rates of homolog screening (HS) and internal network (IN) using 100 ug/L PFAS standard sample. **c** The number of false positive features and false negative features of internal network, FluoroMatch, FindPFAS_1 with $CF_2$, FindPFAS_2 with $CF_2$, $C_2F_4$, and HF, and FindPFAS_3 with $CF_2$, $C_2F_4$, HF, $CF_3$, and $CF_2O$, the diagnostic fragment module of FindPFΔS. Source data are provided as a Source Data file.

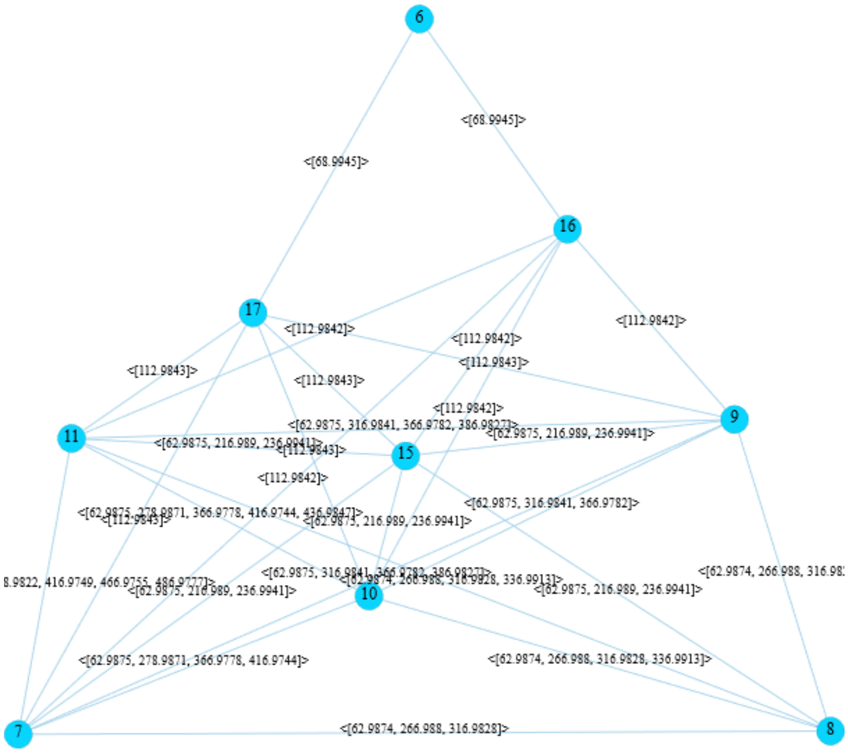


**Supplementary Figure 2. An example of the internal network class.** The nodes represent the homolog in the class and the edge represent to the spectra similarity between nodes. The label of the edge represent the comon fragments between nodes.

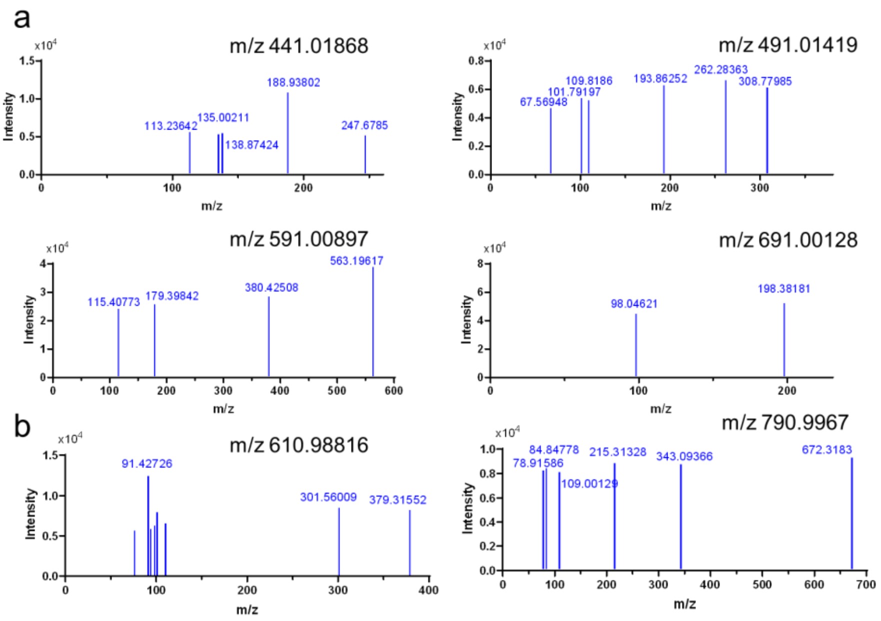


**Supplementary Figure 3. Examples of classes that pass the homolog screening but do not pass internal network. a** Four spectra of the class m/z 441.018868, 491.01419, 591.00897,691.00128 Da.; **b** Two spectra of the class m/z 610.98816, 790.9967 Da.

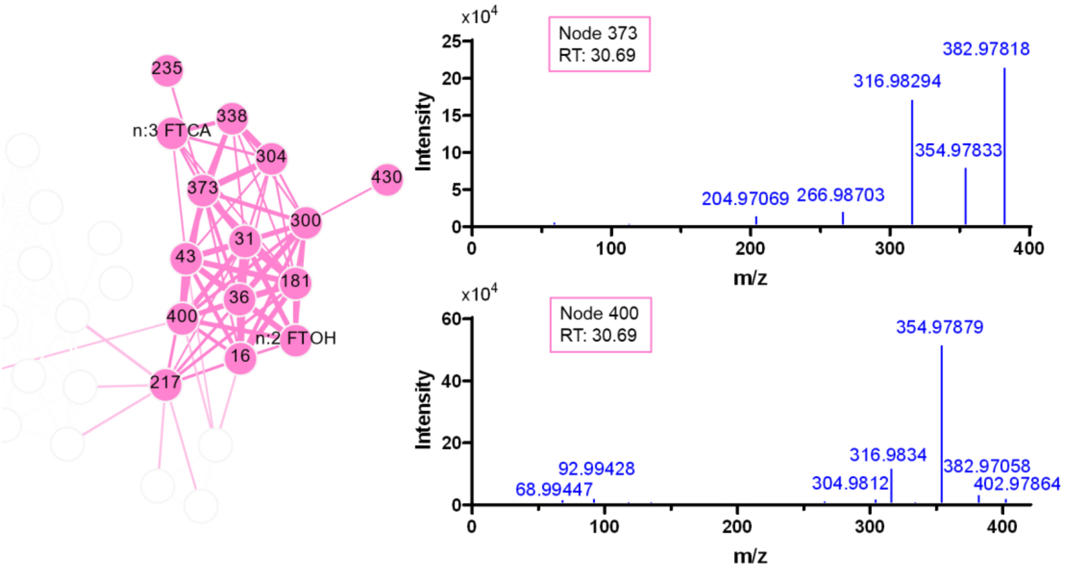


**Supplementary Figure 4. Examples of in-source fragments of Node 181.**

a

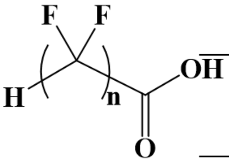


| Product ions formula | Theoretical product ions | Observed product ions | Absolute error | Relative error |
|---|---|---|---|---|
| | | | mDa | ppm |
| C4F7- | 180.98937 | 180.98798 | 7.64 | 1.39 |
| C7F13- | 330.97979 | 330.97971 | 0.25 | 0.08 |

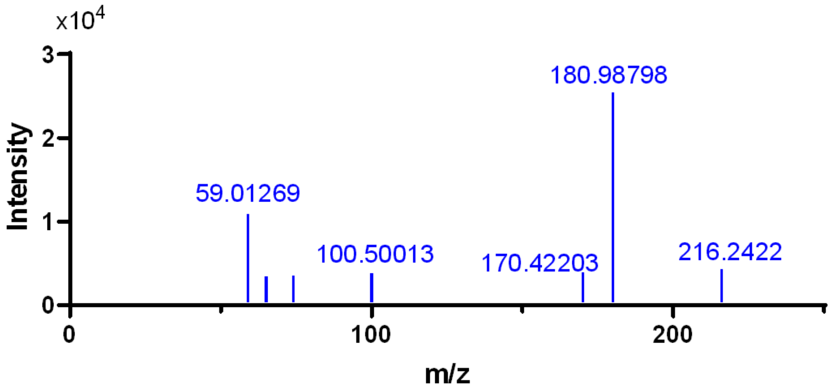


b

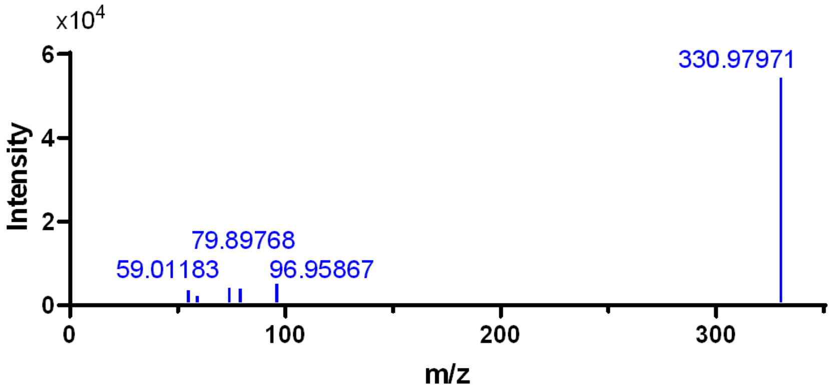


**Supplementary Figure 5. MS/MS spectrum of H-PFCAs. a** MS/MS spectrum of H-PFOA; **b** MS/MS spectrum of H-PFNA.

a

| Product ions formula | Theoretical product ions | Observed product ions | Absolute error | Relative error |
| --- | --- | --- | --- | --- |
| | | | mDa | ppm |
| CFO2- | 62.98878 | 62.98751 | 1.27 | 20.20 |
| C7F7- | 216.98937 | 216.98906 | 0.31 | 1.44 |
| C9F11- | 316.98298 | 316.98413 | 1.14 | 3.61 |
| C10F13- | 366.97979 | 366.97818 | 1.61 | 4.39 |
| C10HF14- | 386.98602 | 386.98273 | 3.29 | 8.50 |

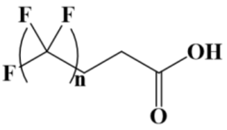


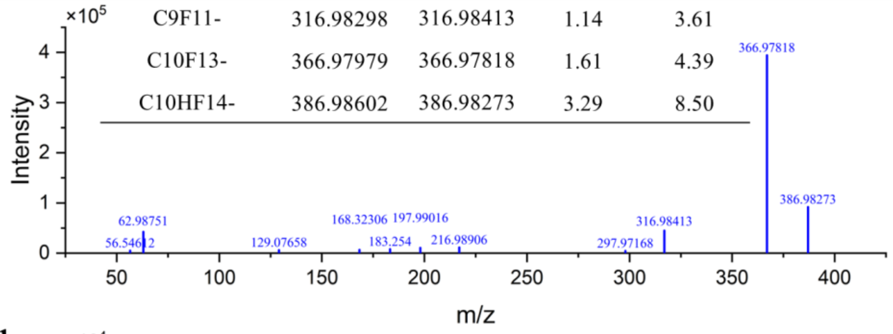


b

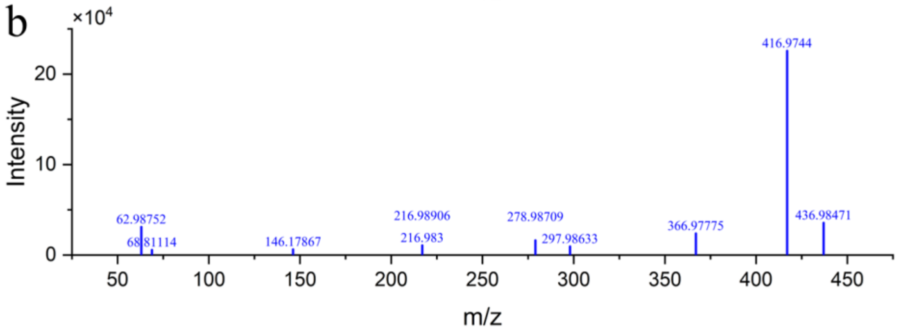


**Supplementary Figure 6. MS/MS spectrum of n:3 FTCAs. a** MS/MS spectrum of 8:3 FTCA; **b** MS/MS spectrum of 9:3 FTCA.

a

| Product ions formula | Theoretical product ions | Observed product ions | Absolute error | Relative error |
|---|---|---|---|---|
| | | | mDa | ppm |
| C2F5- | 118.99257 | 118.99209 | 0.48 | 4.00 |

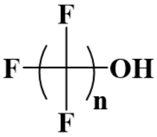


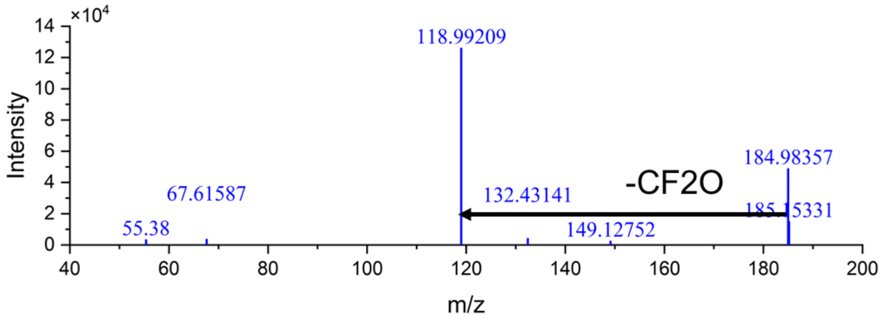


b

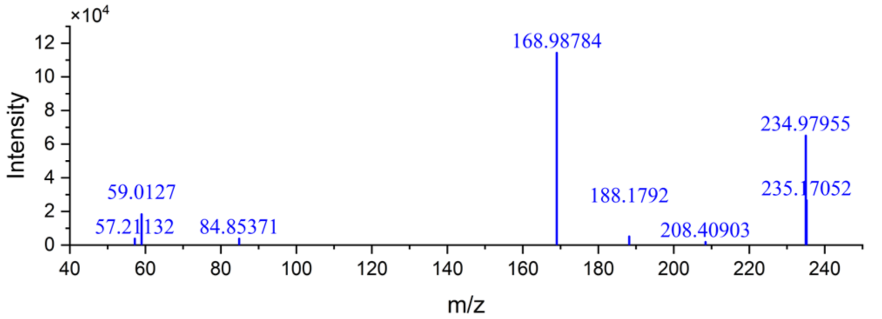


**Supplementary Figure 7. MS/MS spectrum of PAs. a** MS/MS spectrum of PAs n=3; **b** MS/MS spectrum of PAs n=4.

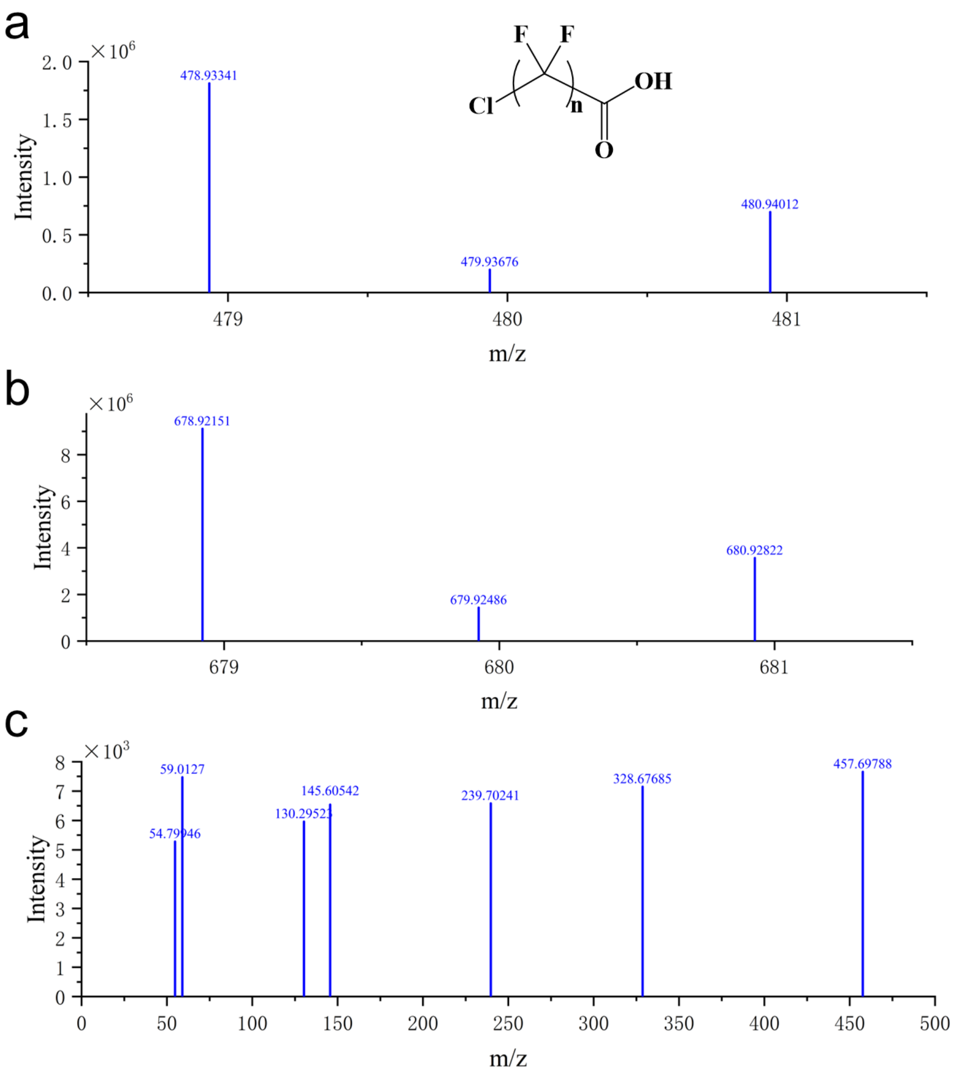


**Supplementary Figure 8. Spectrum of Cl-PFCAs. a** MS spectrum of Cl-PFNA; **b** MS spectrum of Cl-PFTrDA; **c** MS/MS spectrum of Cl-PFNA.

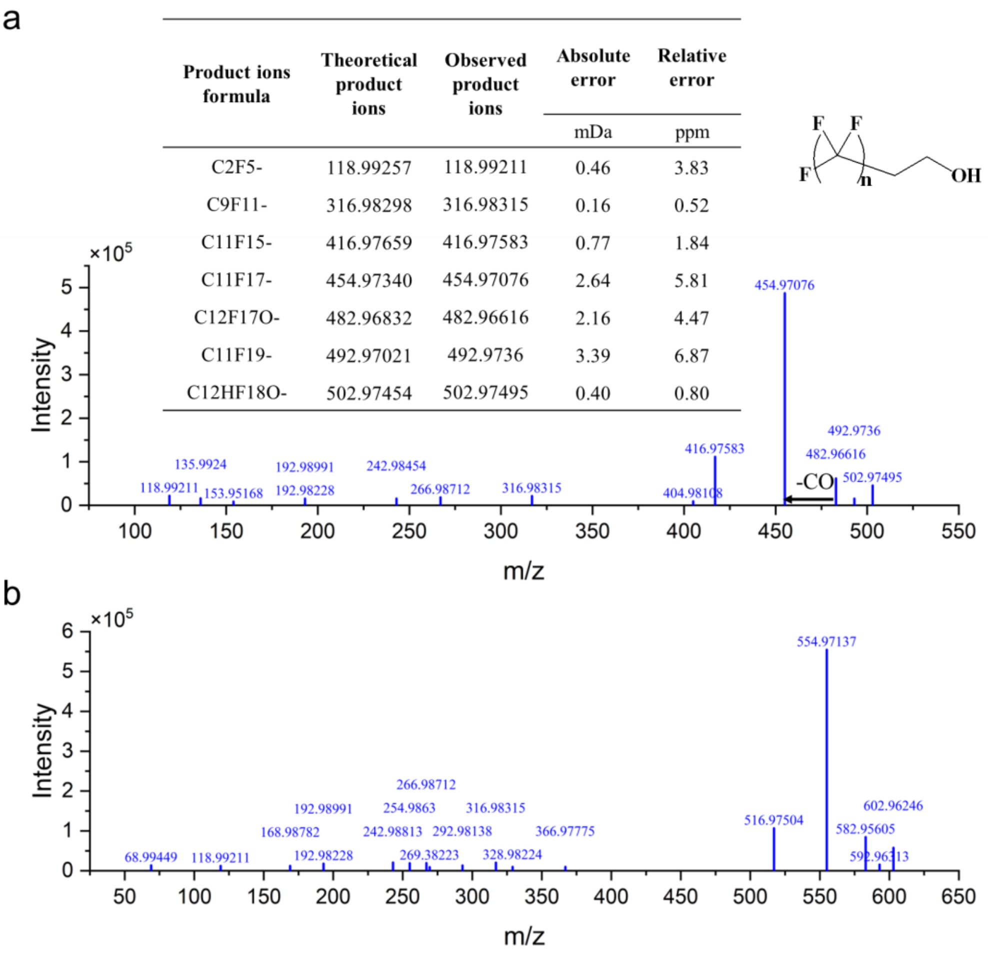


| Product ions formula | Theoretical product ions | Observed product ions | Absolute error | Relative error |
|---|---|---|---|---|
| | | | mDa | ppm |
| C2F5- | 118.99257 | 118.99211 | 0.46 | 3.83 |
| C9F11- | 316.98298 | 316.98315 | 0.16 | 0.52 |
| C11F15- | 416.97659 | 416.97583 | 0.77 | 1.84 |
| C11F17- | 454.97340 | 454.97076 | 2.64 | 5.81 |
| C12F17O- | 482.96832 | 482.96616 | 2.16 | 4.47 |
| C11F19- | 492.97021 | 492.9736 | 3.39 | 6.87 |
| C12HF18O- | 502.97454 | 502.97495 | 0.40 | 0.80 |

**Supplementary Figure 9. MS/MS spectrum of n:2 FTOHs. a** MS/MS spectrum of 10:2 FTOH; **b** MS/MS spectrum of 12:2 FTOH.

a

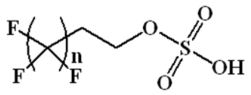


| Product ions formula | Theoretical product ions | Observed product ions | Absolute error | Relative error |
|---|---|---|---|---|
| | | | mDa | ppm |
| SO4H- | 96.9601 | 96.95865 | 1.45 | 15 |

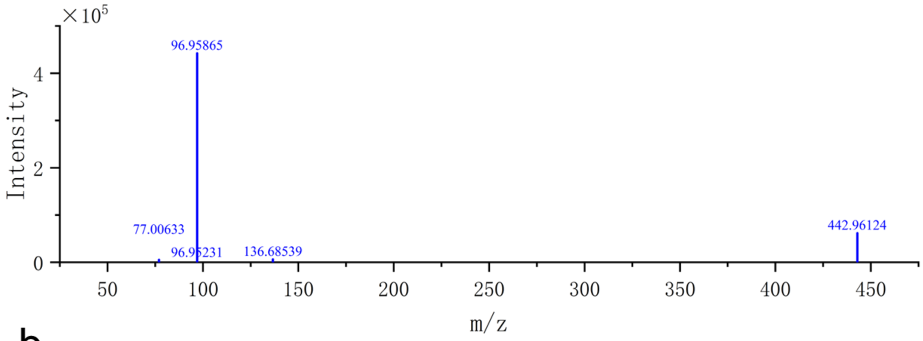


b

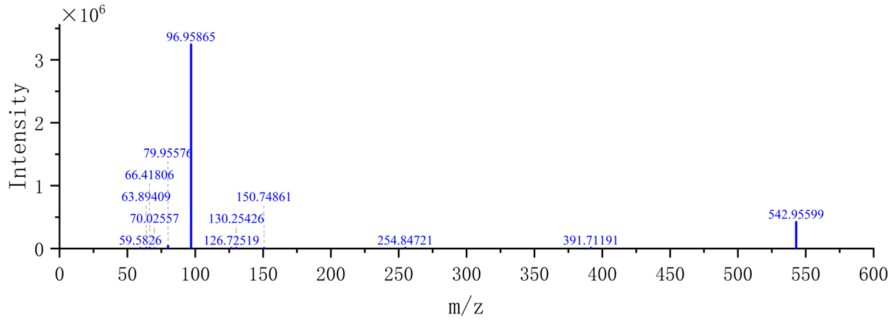


**Supplementary Figure 10. MS/MS spectrum of n:2 FTSFs. a** MS/MS spectrum of 8:2 FTSF; **b** MS/MS spectrum of 10:2 FTSF.

a

| Product ions formula | Theoretical product ions | Observed product ions | Absolute error | Relative error |
|---|---|---|---|---|
| | | | mDa | ppm |
| CF3- | 68.99576 | 68.99439 | 1.37 | 19.85 |
| C14H8F3O2- | 265.04818 | 265.04889 | 0.70 | 2.64 |

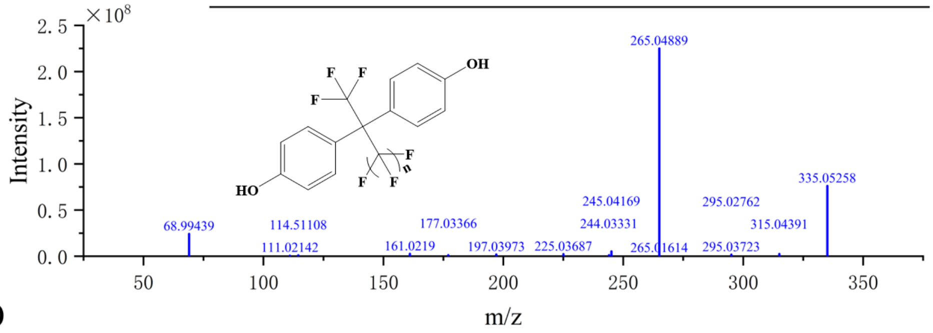


b

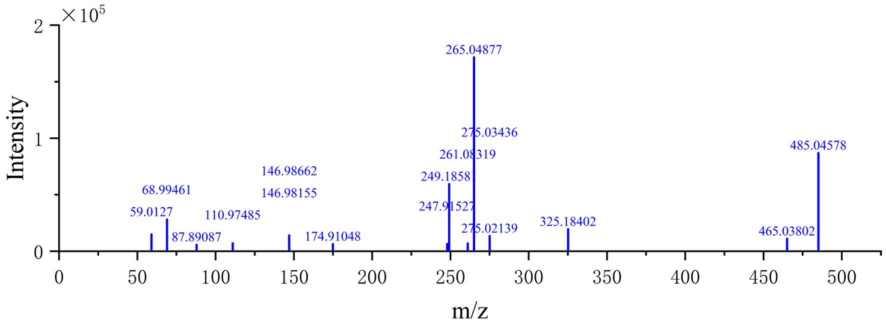


c

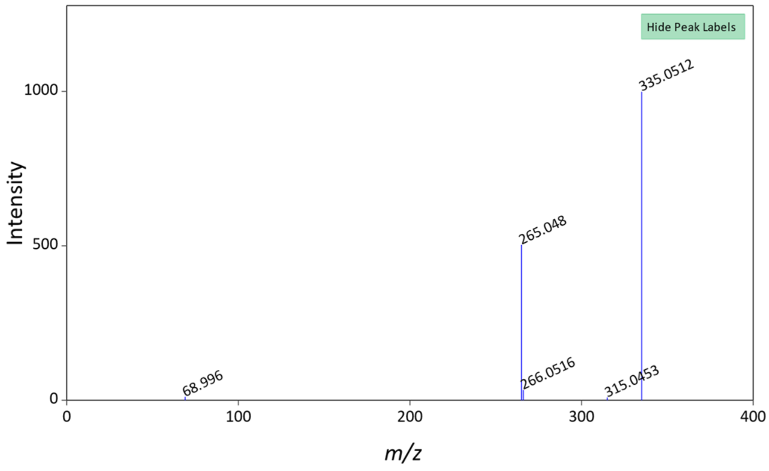


**Supplementary Figure 11. MS/MS spectrum of BPAFs. a** MS/MS spectrum of BPAF in samples. **b** MS/MS spectrum of C18-BPAF in samples. **c** MS/MS spectrum of BPAF in MassBank.

a

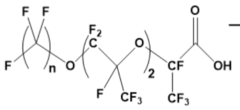


| Product ions formula | Theoretical product ions | Observed product ions | Absolute error | Relative error |
|---|---|---|---|---|
| | | | mDa | ppm |
| C2F5O- | 134.98748 | 134.98726 | 0.22 | 1.64 |
| C5F11O2- | 300.97281 | 300.97479 | 1.98 | 6.56 |
| C8F17O3- | 466.95814 | 466.95529 | 2.86 | 6.12 |

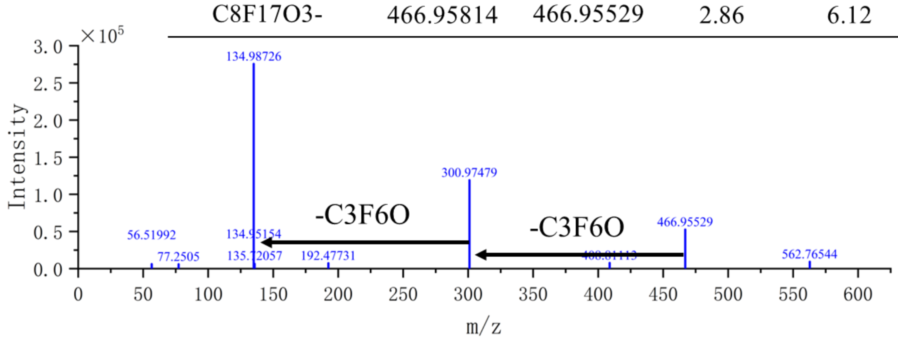


b

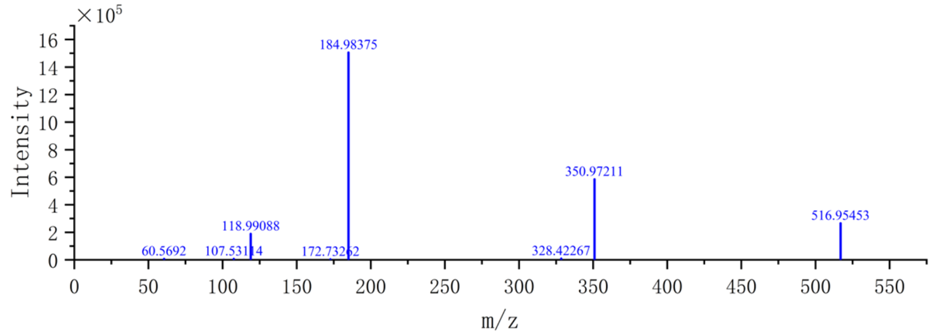


**Supplementary Figure 12. MS/MS spectrum of PFTrECAs. a** MS/MS spectrum of C11 PFTrECA; **b** MS/MS spectrum of C12 PFTrECA.

a

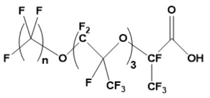


| Product ions formula | Theoretical product ions | Observed product ions | Absolute error | Relative error |
|---|---|---|---|---|
| | | | mDa | ppm |
| C2F5O- | 134.98748 | 134.9872 | 0.28 | 2.08 |
| C5F11O2- | 300.97281 | 300.97476 | 1.95 | 6.46 |
| C8F17O3- | 466.95814 | 466.95605 | 2.10 | 4.49 |
| C11F23O4- | 632.94348 | 632.94885 | 5.37 | 8.48 |

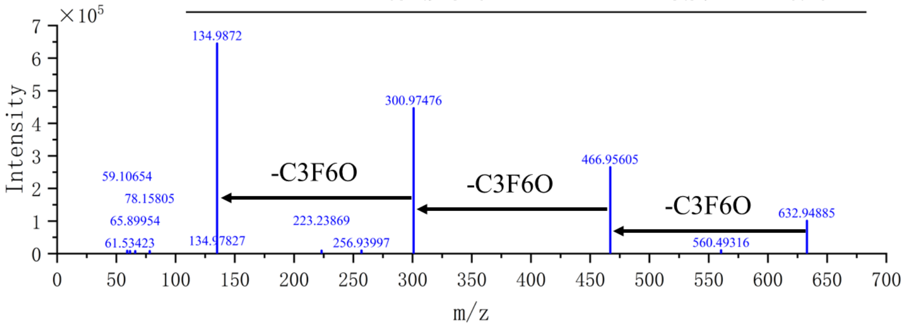


b

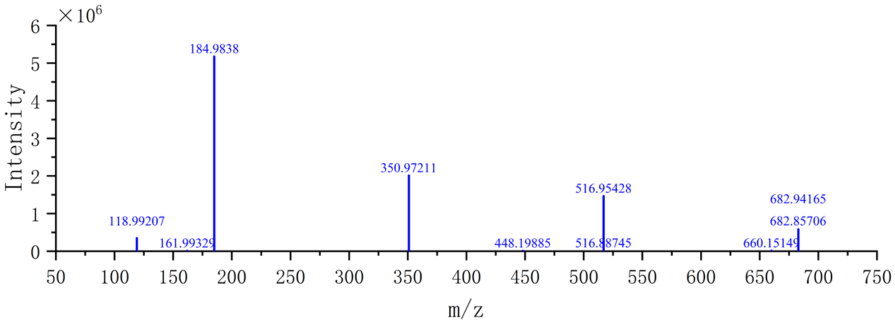


**Supplementary Figure 13. MS/MS spectrum of PFTeECAs. a** MS/MS spectrum of C14-PFTeECA; **b** MS/MS spectrum of C15-PFTeECA.

a

| Product ions formula | Theoretical product ions | Observed product ions | Absolute error | Relative error |
|---|---|---|---|---|
| | | | mDa | ppm |
| C2F5O- | 134.98748 | 134.98712 | 0.36 | 2.67 |
| C5F11O2- | 300.97281 | 300.97488 | 2.06 | 6.86 |
| C8F17O3- | 466.95814 | 466.95639 | 1.76 | 3.78 |
| C11F23O4- | 632.94348 | 632.95007 | 6.59 | 10.41 |
| C14F26O5- | 798.92881 | 798.93579 | 6.97 | 8.73 |

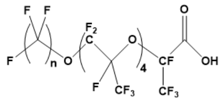

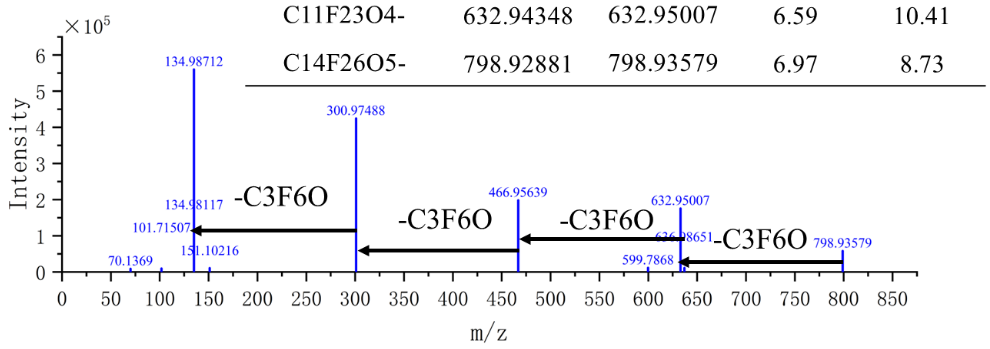


b

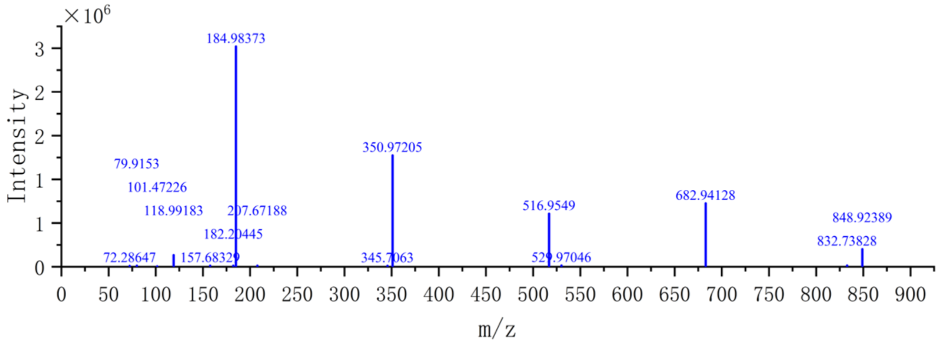


**Supplementary Figure 14. MS/MS spectrum of PFPeECAs. a** MS/MS spectrum of C17-PFPeECA; **b** MS/MS spectrum of C18-PFPeECA.

a

| Product ions formula | Theoretical product ions | Observed product ions | Absolute error | Relative error |
|---|---|---|---|---|
| | | | mDa | ppm |
| C3H5O3- | 89.02442 | 89.0231 | 1.32 | 14.82 |
| C7F7- | 216.98937 | 216.98891 | 0.46 | 2.13 |
| C7F9- | 254.98617 | 254.98576 | 0.42 | 1.64 |
| C8F9O- | 282.98019 | 282.98038 | 0.71 | 2.52 |
| C7F11- | 292.98298 | 292.98572 | 2.73 | 9.33 |
| C8HF10O- | 302.98732 | 302.98517 | 2.15 | 7.10 |
| C8H2F11O- | 322.99355 | 322.99188 | 1.67 | 5.17 |

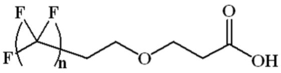


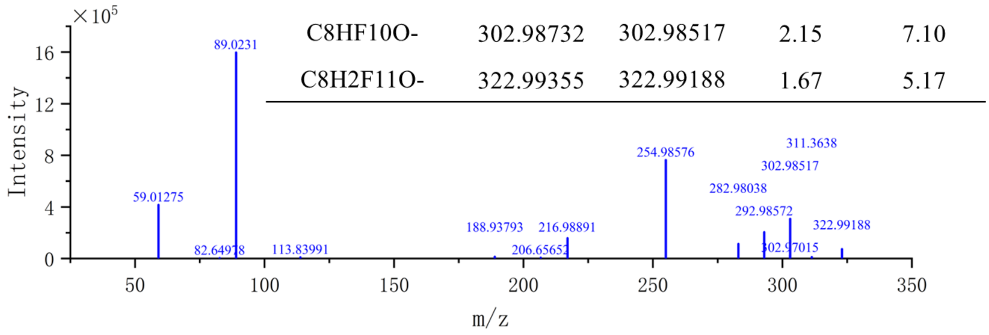


b

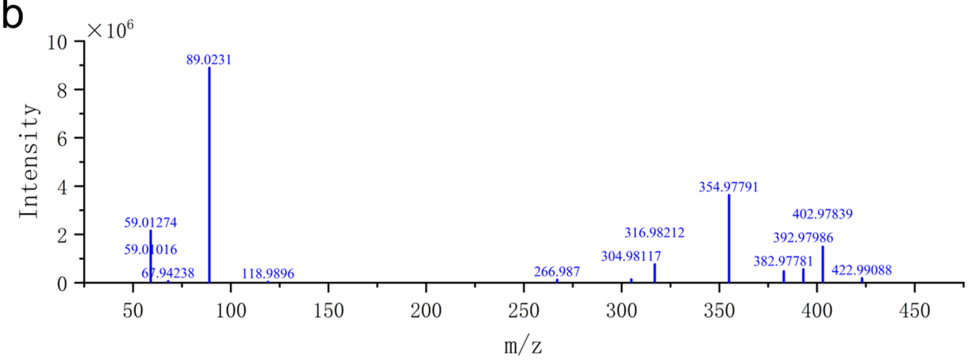


**Supplementary Figure 15. MS/MS spectrum of n:2:3 PFECAs. a** MS/MS spectrum of 6:2:3 PFECA; **b** MS/MS spectrum of 8:2:3 PFECA.

a

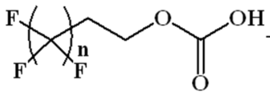


| Product ions formula | Theoretical product ions | Observed product ions | Absolute error | Relative error |
|---|---|---|---|---|
| | | | mDa | ppm |
| C3H3O3- | 87.00877 | 87.00758 | 1.19 | 13.66 |
| C7F9- | 254.98617 | 254.9863 | 0.23 | 0.99 |

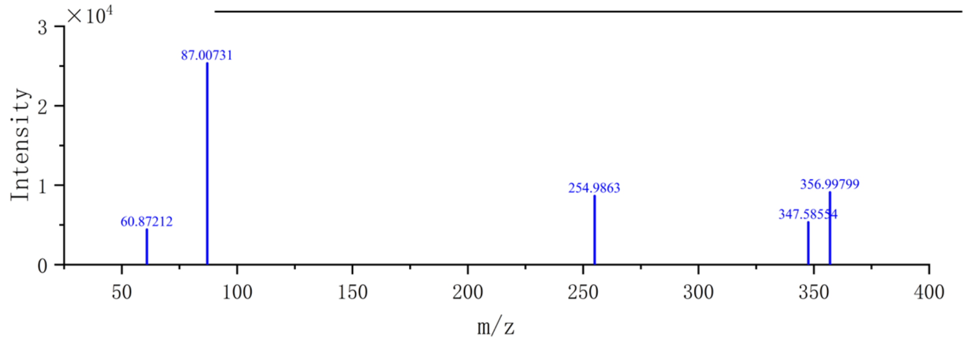


b

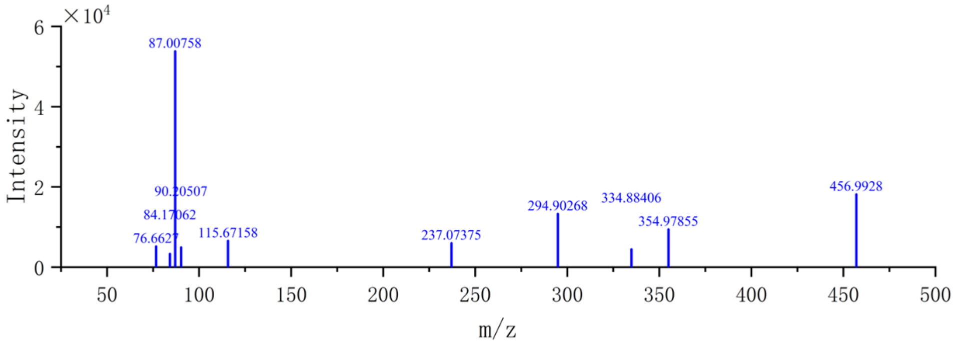


**Supplementary Figure 16. MS/MS spectrum of n:3 PFECAs. a** MS/MS spectrum of 5:3 PFECA; **b** MS/MS spectrum of 7:3 PFECA.

a

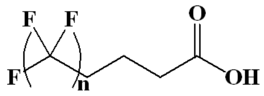


| Product ions formula | Theoretical product ions | Observed product ions | Absolute error | Relative error |
|---|---|---|---|---|
| | | | mDa | ppm |
| FCO2- | 62.98878 | 62.98739 | 1.39 | 22.11 |
| C9F7- | 240.98937 | 240.98845 | 0.92 | 3.83 |
| C9HF8- | 260.99560 | 260.99594 | 0.34 | 1.30 |
| C9H2F9- | 281.00182 | 281.00034 | 1.49 | 5.30 |

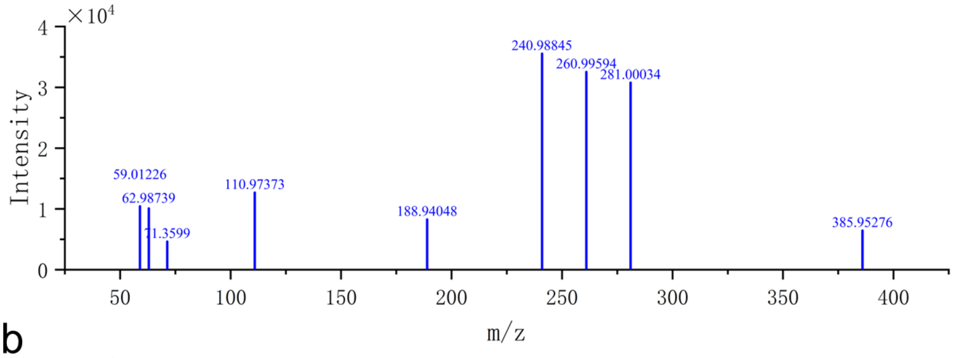


b

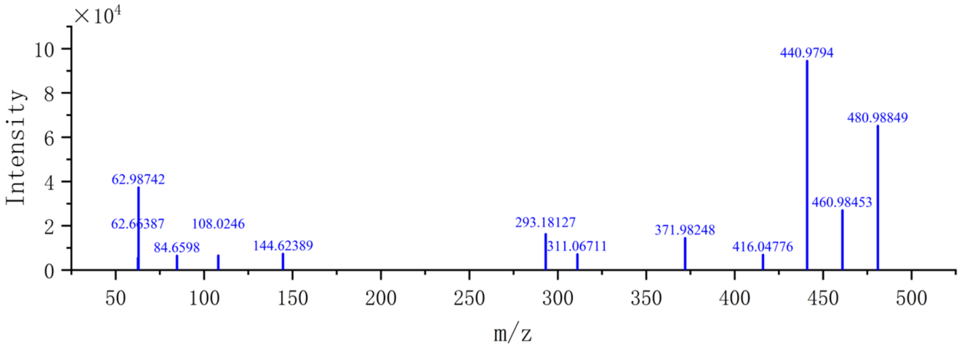


**Supplementary Figure 17. MS/MS spectrum of n:4 FTCAs. a** MS/MS spectrum of 6:4 FTCA; **b** MS/MS spectrum of 10:4 FTCA.

a

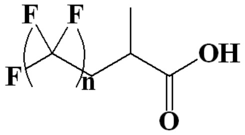


| Product ions formula | Theoretical product ions | Observed product ions | Absolute error | Relative error |
|---|---|---|---|---|
| | | | mDa | ppm |
| C4H5O2- | 85.02950 | 85.02827 | 1.23 | 14.51 |
| C3F7- | 168.98937 | 168.98802 | 1.35 | 8.00 |
| C4F9- | 218.98617 | 218.98566 | 0.52 | 2.37 |
| C8F17- | 418.97340 | 418.97638 | 2.98 | 7.10 |

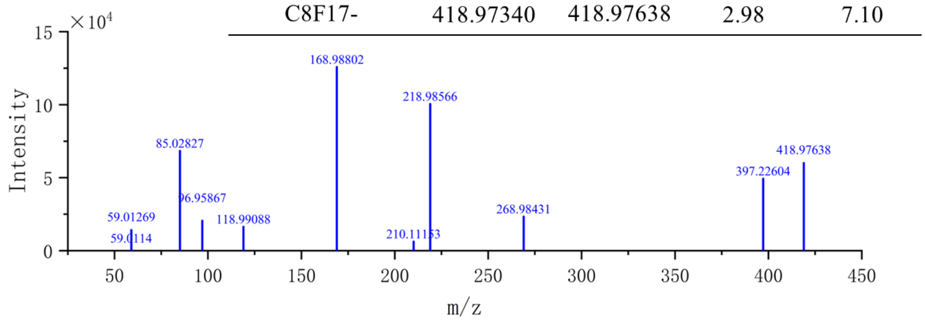


b

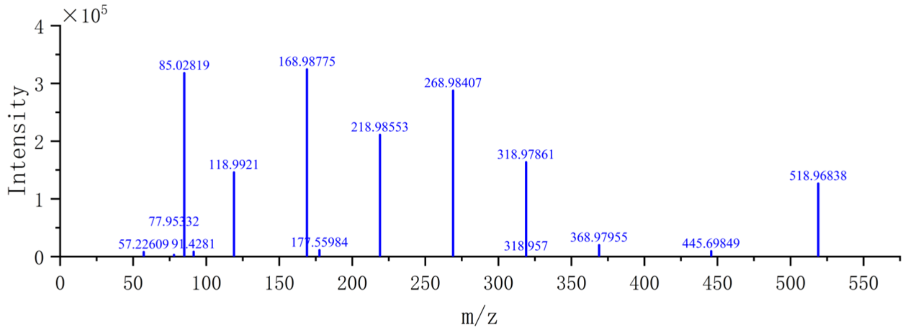


**Supplementary Figure 18. MS/MS spectrum of m-n:4 FTCAs. a** MS/MS spectrum of m-8:4 FTCA; **b** MS/MS spectrum of m-10:4 FTCA.

a

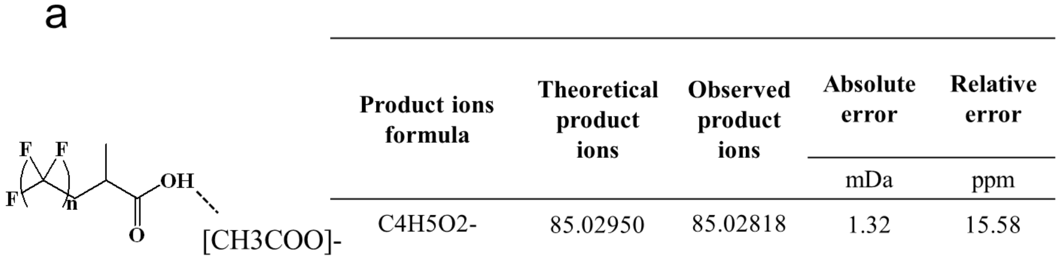


| Product ions formula | Theoretical product ions | Observed product ions | Absolute error | Relative error |
|---|---|---|---|---|
| | | | mDa | ppm |
| C4H5O2- | 85.02950 | 85.02818 | 1.32 | 15.58 |
| C3F7- | 168.98937 | 168.98798 | 1.39 | 8.24 |
| C4F9- | 218.98617 | 218.98582 | 0.36 | 1.64 |
| C8F17- | 418.97340 | 418.97684 | 3.44 | 8.20 |

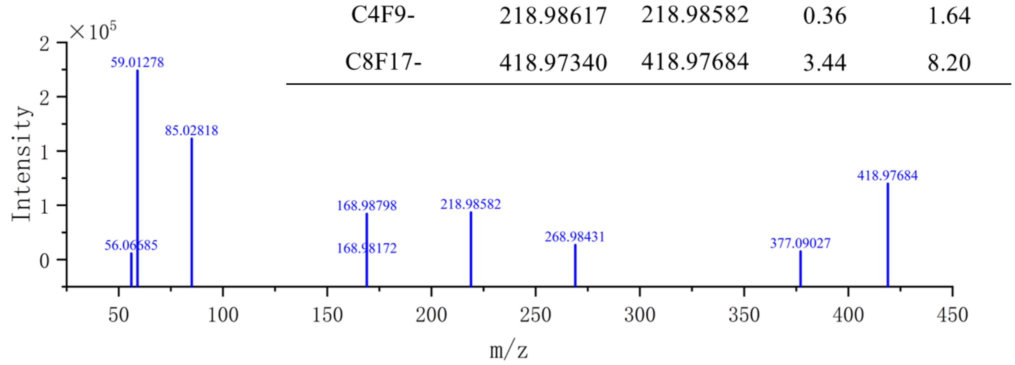


b

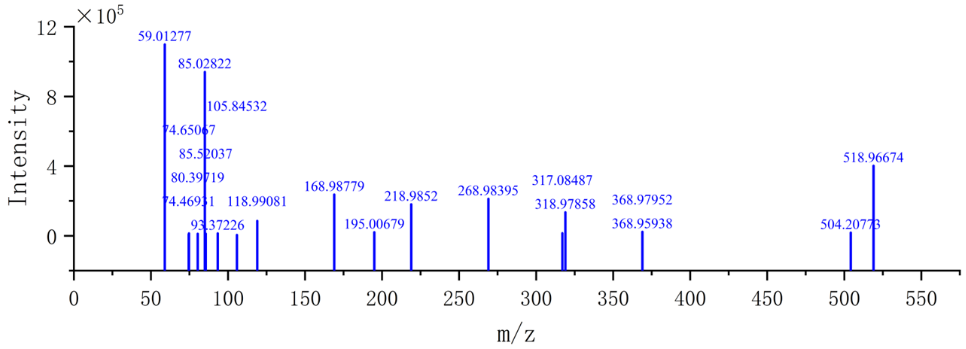


**Supplementary Figure 19. MS/MS spectrum of adducts of m-n:4 FTCAs. a** MS/MS spectrum of acetic acid adduct of m-8:4 FTCA; **b** MS/MS spectrum of the acetic acid adduct of m-10:4 FTCA.

a

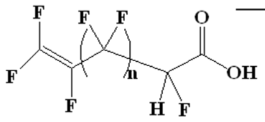


| Product ions formula | Theoretical product ions | Observed product ions | Absolute error | Relative error |
|---|---|---|---|---|
| | | | mDa | ppm |
| C3F3- | 92.99576 | 92.99432 | 1.44 | 15.48 |
| C6F9- | 242.98617 | 242.98502 | 1.16 | 4.77 |
| C6HF10- | 262.99240 | 262.99417 | 1.76 | 6.70 |

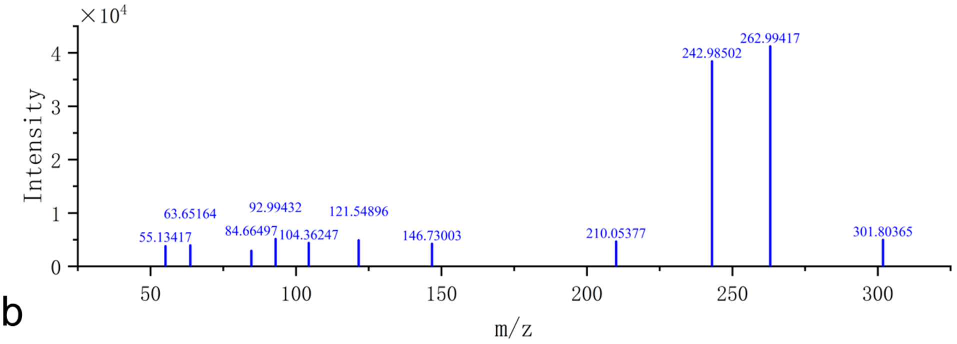


b

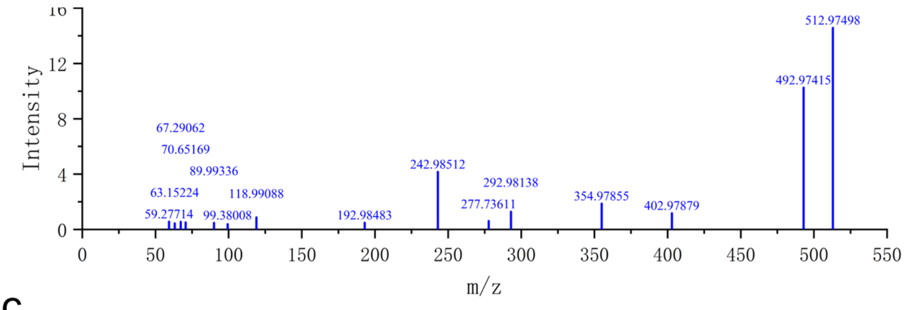


c

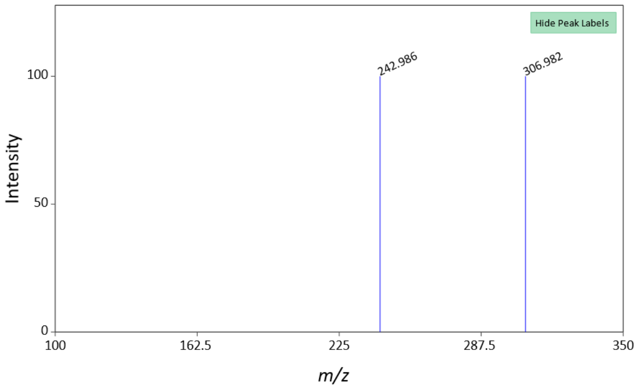


**Supplementary Figure 20. MS/MS spectrum of dH-PFCAs. a** MS/MS spectrum of dH-PFHpA; **b** MS/MS spectrum of dH-PFDoA. (C) MS/MS spectrum of dH-PFHpA in MassBank.

a

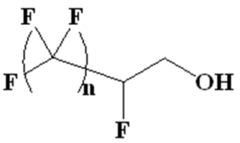


| Product ions formula | Theoretical product ions | Observed product ions | Absolute error | Relative error |
|---|---|---|---|---|
| | | | mDa | ppm |
| C3F5- | 130.99256 | 130.99179 | 0.78 | 5.92 |
| C4F7- | 180.98937 | 180.98827 | 1.10 | 6.09 |
| C6F7- | 204.98937 | 204.98984 | 0.47 | 2.28 |
| C6F9- | 242.98617 | 242.98454 | 1.64 | 6.75 |
| C7F9O- | 270.98109 | 270.98328 | 2.19 | 8.07 |
| C7HF10O- | 290.98732 | 290.98813 | 0.81 | 2.78 |
| C7H2F11O- | 310.99355 | 310.99493 | 1.38 | 4.44 |

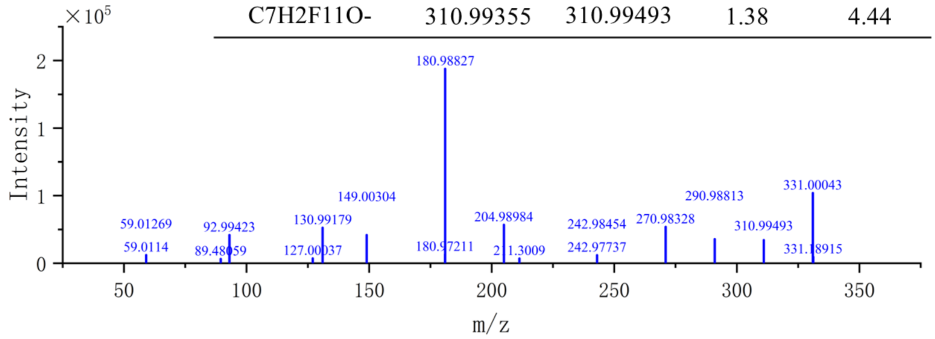


b

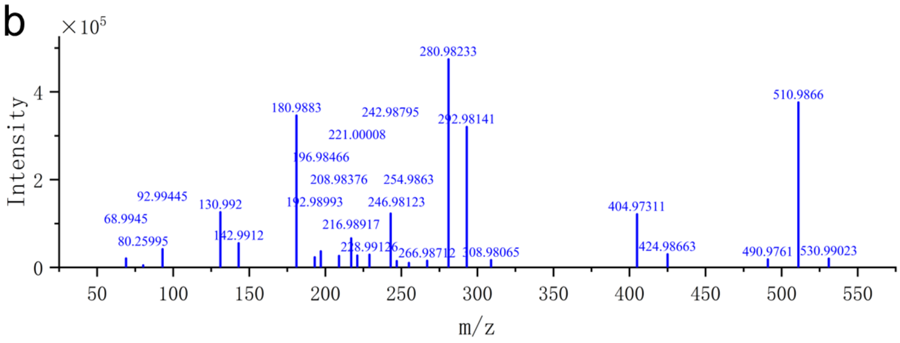


**Supplementary Figure 21. MS/MS spectrum of H-n:1 FTOHs. a** MS/MS spectrum of H-6:1 FTOH; **b** MS/MS spectrum of H-10:1 FTOH.

a

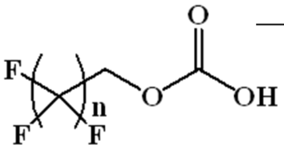


| Product ions formula | Theoretical product ions | Observed product ions | Absolute error | Relative error |
|---|---|---|---|---|
| | | | mDa | ppm |
| C5F7- | 192.98937 | 192.98991 | 0.54 | 2.78 |
| C5F9- | 230.98617 | 230.98595 | 0.23 | 0.99 |
| C7F13- | 330.97979 | 330.97818 | 1.61 | 4.87 |
| C8F13O- | 358.97470 | 358.9765 | 1.79 | 5.00 |

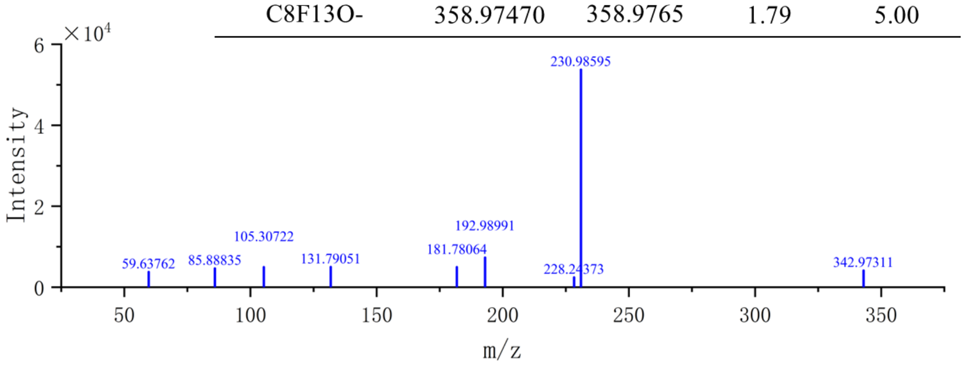


b

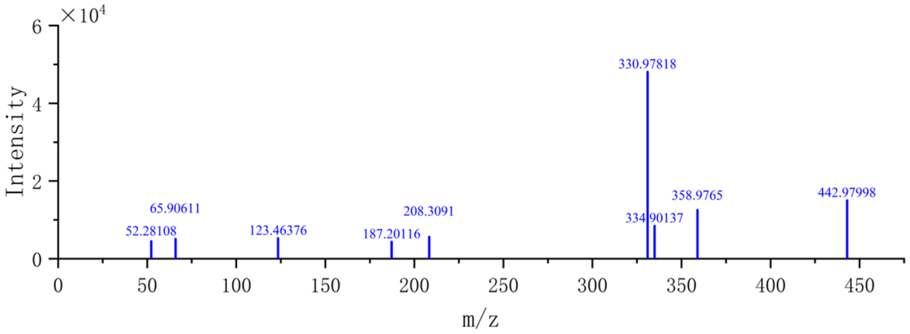


**Supplementary Figure 22. MS/MS spectrum of n:2 PFECAs. a** MS/MS spectrum of 5:2 PFECA; **b** MS/MS spectrum of 7:2 PFECA.

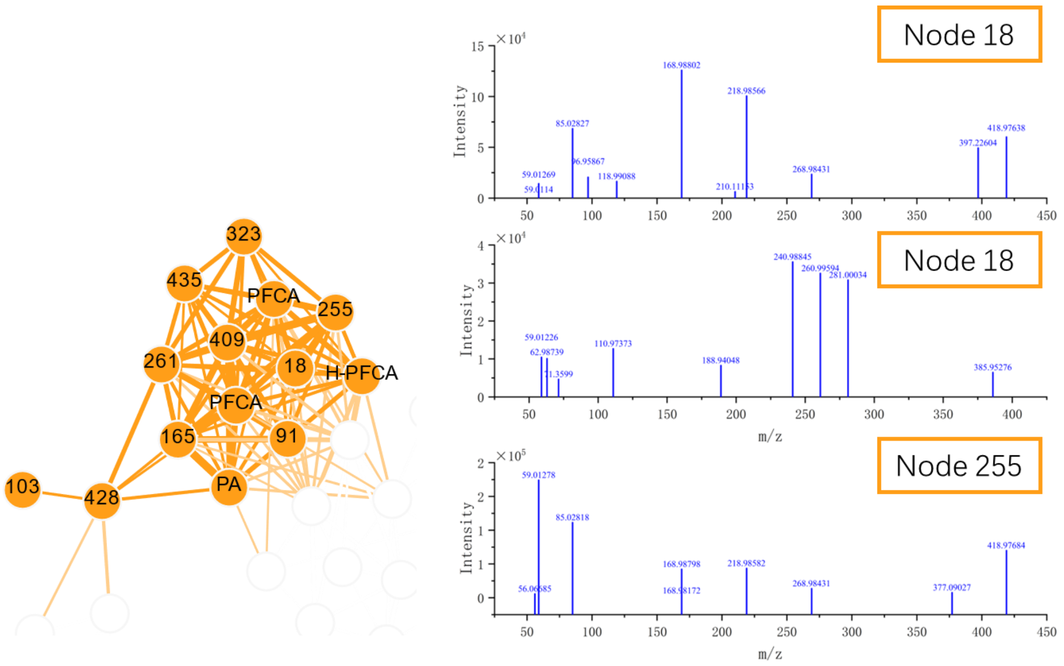


**Supplementary Figure 23. Spectra of classes of community c.** The nodes refer to merge spectrum of classes. The edges refer to the spectra similarity between nodes.

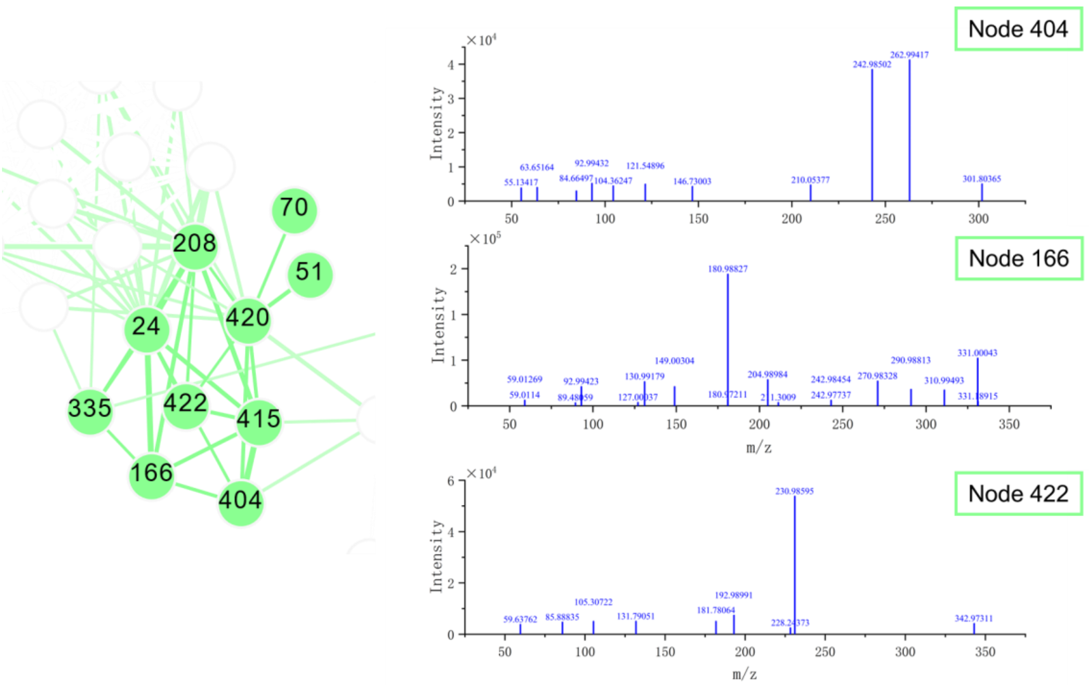


**Supplementary Figure 24. Spectra of classes of community d.**

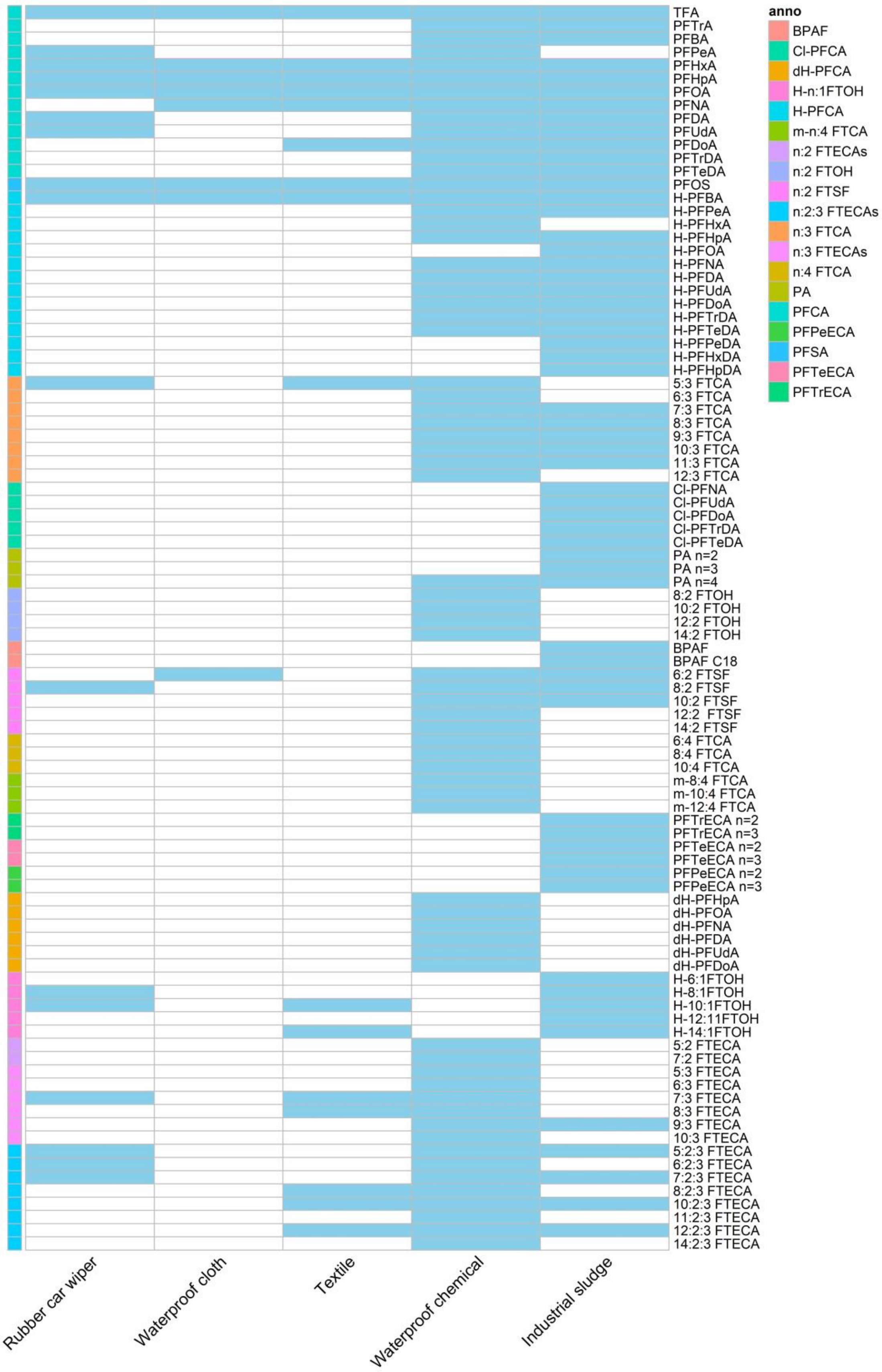


**Supplementary Figure 25. Detection of PFAS in samples.** Blue refers to be detected while white refers to not. Source data are provided as a Source Data file.

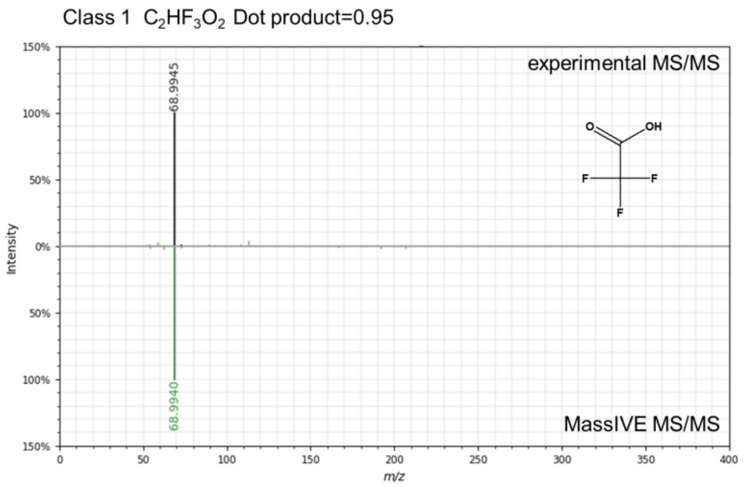


**Supplementary Figure 26. Mirror plot of TFA.**

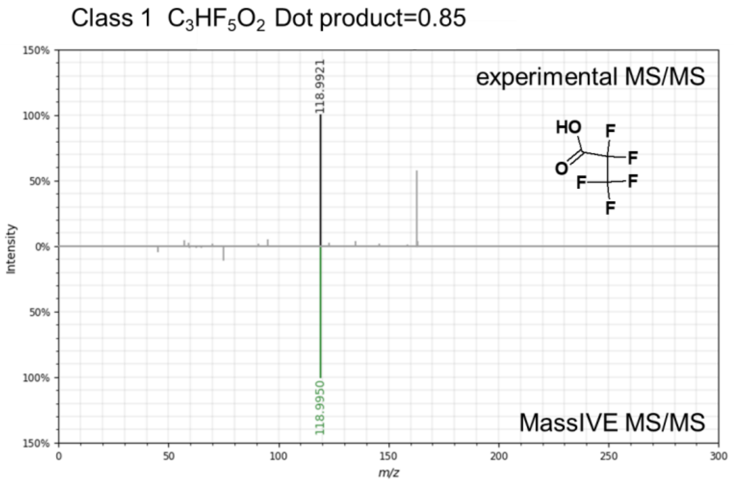


**Supplementary Figure 27. Mirror plot of PFTrA.**

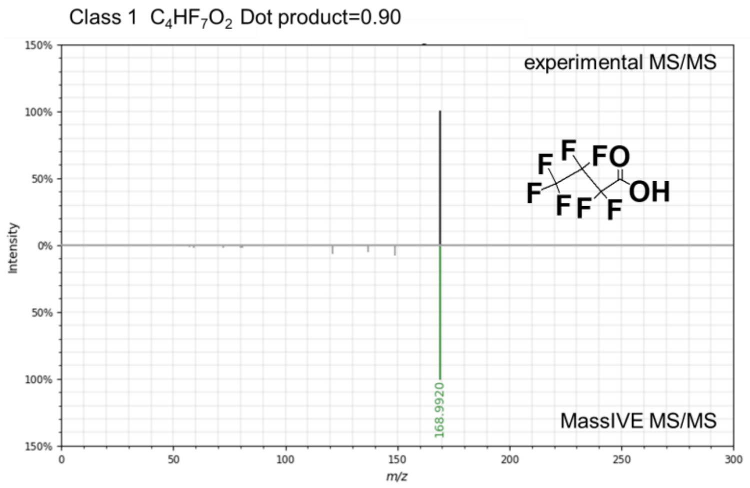


**Supplementary Figure 28. Mirror plot of PFBA.**

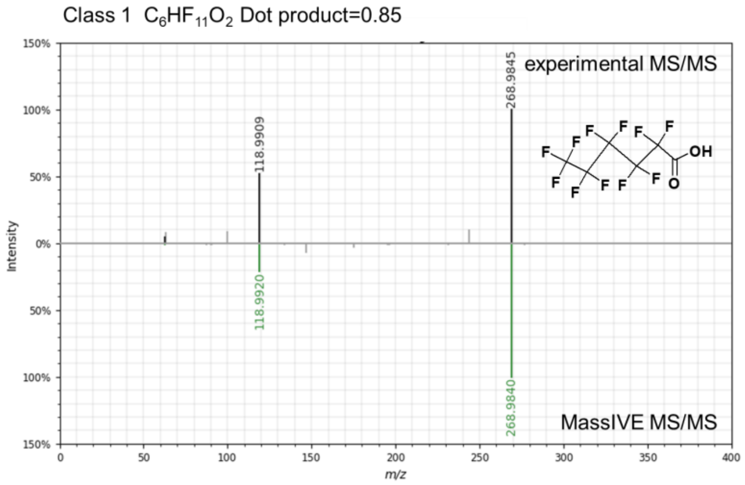


**Supplementary Figure 29. Mirror plot of PFHxA.**

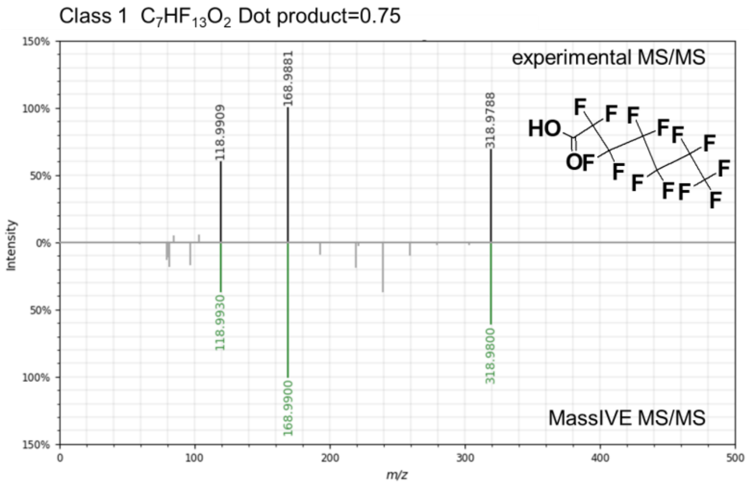


**Supplementary Figure 30. Mirror plot of PFHpA.**

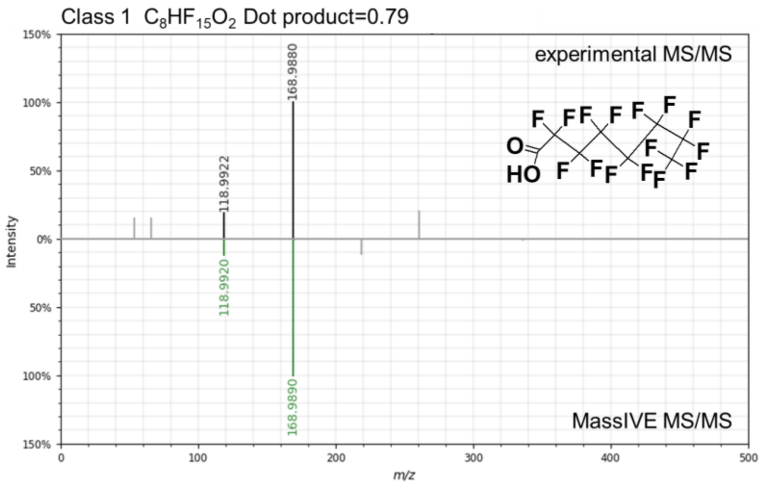


**Supplementary Figure 31. Mirror plot of PFOA.**

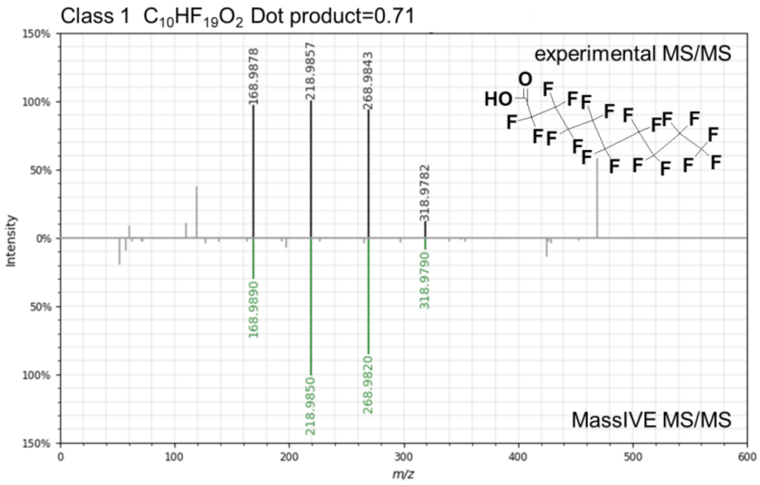


**Supplementary Figure 32. Mirror plot of PFDA.**

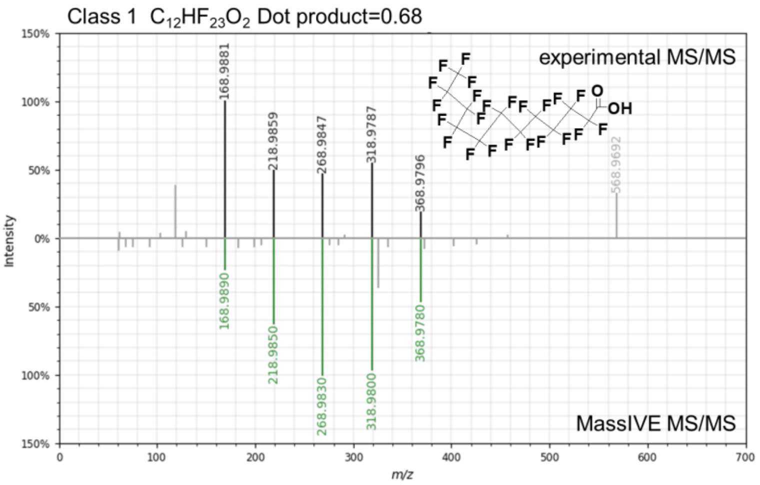


**Supplementary Figure 33. Mirror plot of PFDoA.**

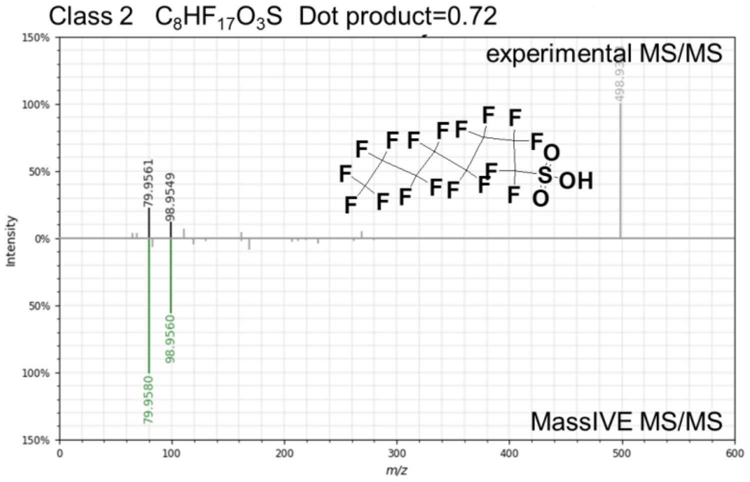


**Supplementary Figure 34. Mirror plot of PFOS.**

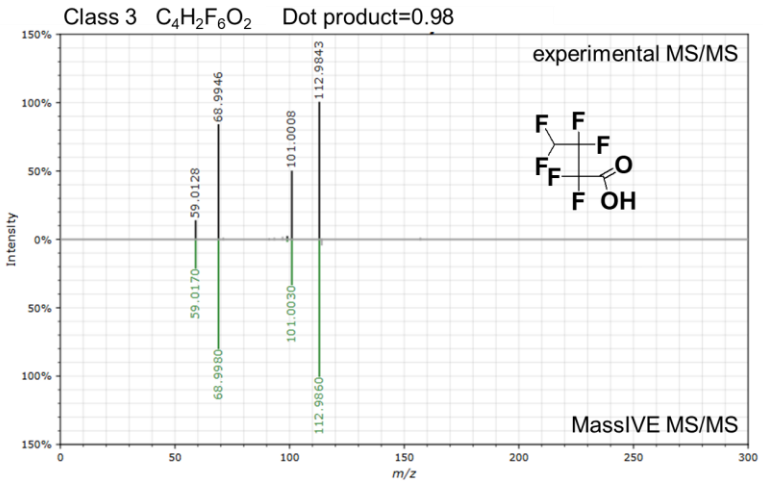


**Supplementary Figure 35. Mirror plot of H-PFBA.**

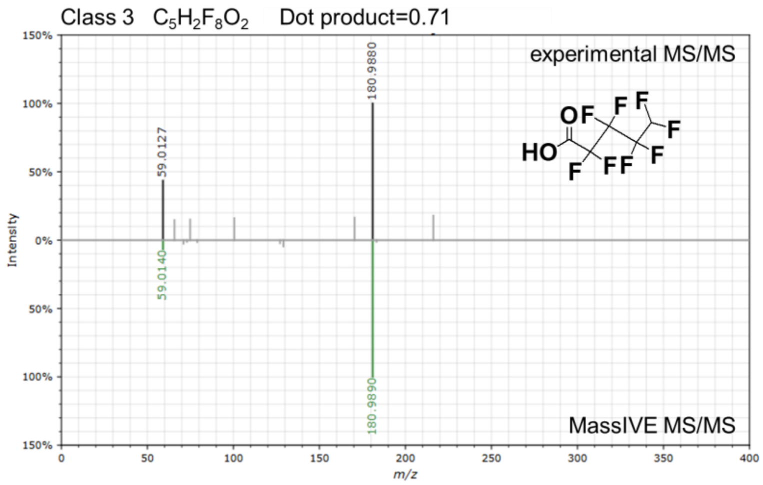


**Supplementary Figure 36. Mirror plot of H-PFPeA.**

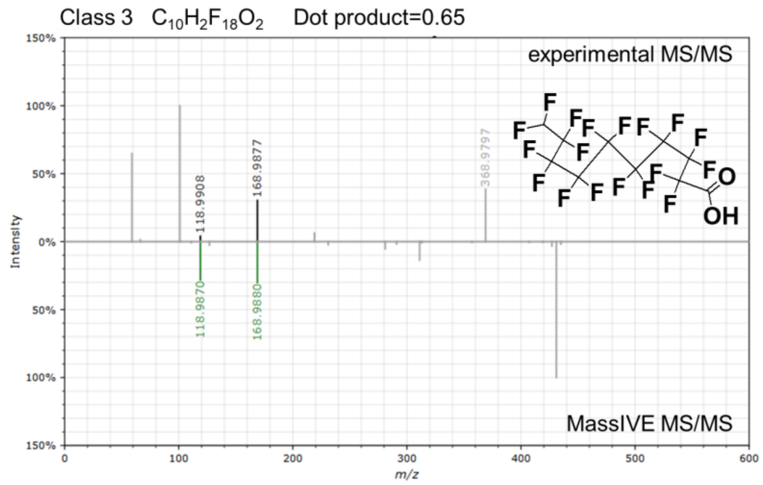


**Supplementary Figure 37. Mirror plot of H-PFDA.**

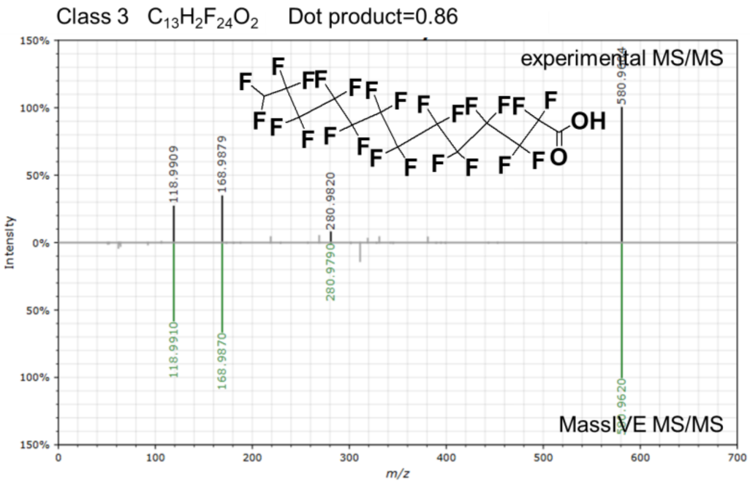


**Supplementary Figure 38. Mirror plot of H-PFTrDA.**

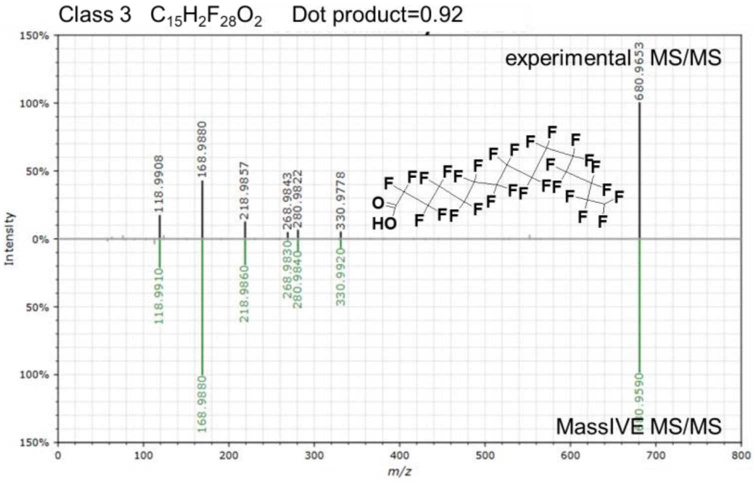


**Supplementary Figure 39. Mirror plot of H-PFPeDA.**

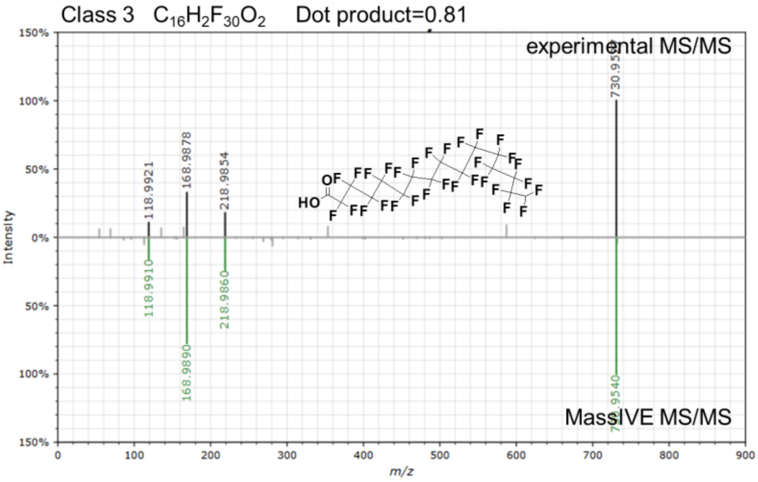


**Supplementary Figure 40. Mirror plot of H-PFHxDA.**

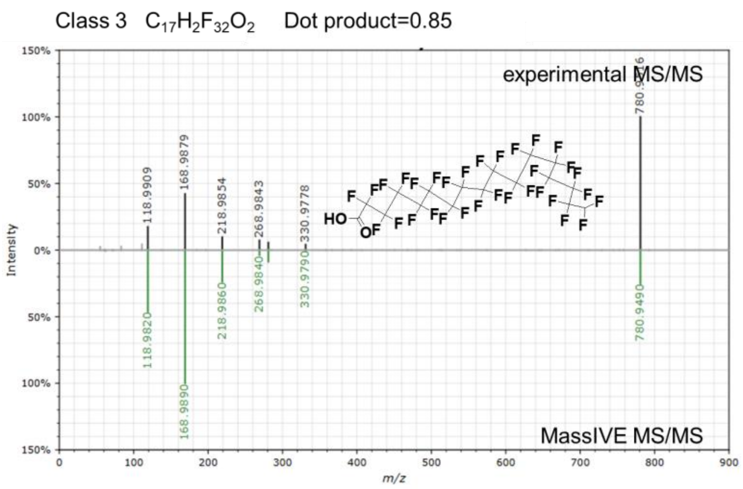


**Supplementary Figure 41. Mirror plot of H-PFHpDA.**

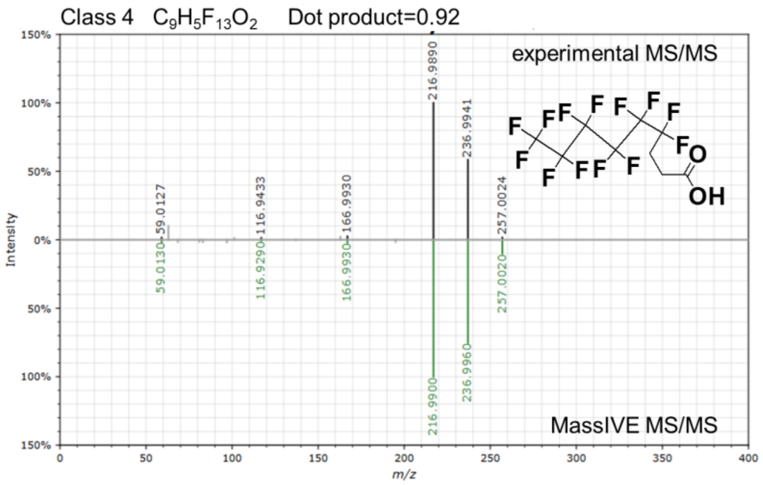


**Supplementary Figure 42. Mirror plot of 6:3 FTCA.**

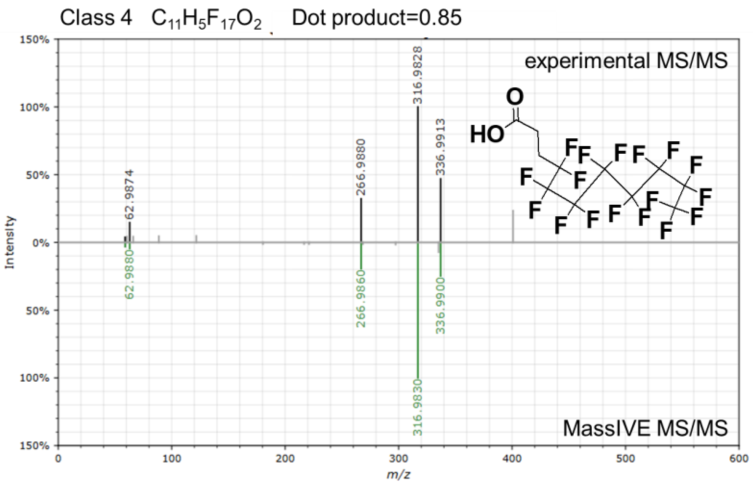


**Supplementary Figure 43. Mirror plot of 8:3 FTCA.**

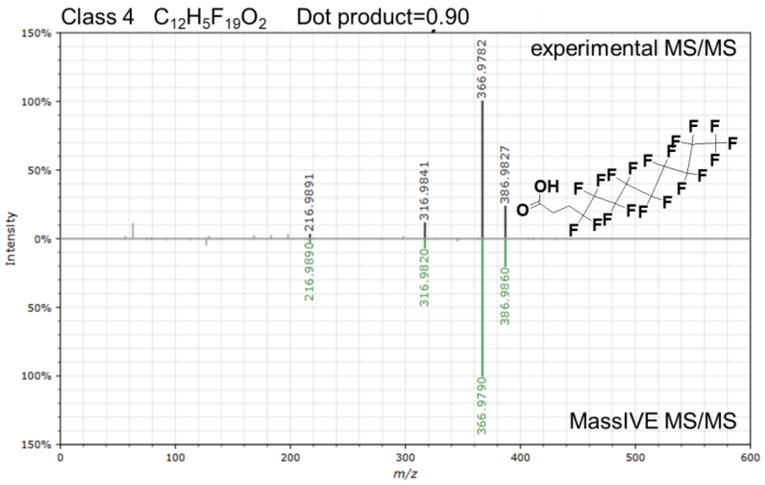


**Supplementary Figure 44. Mirror plot of 9:3 FTCA.**

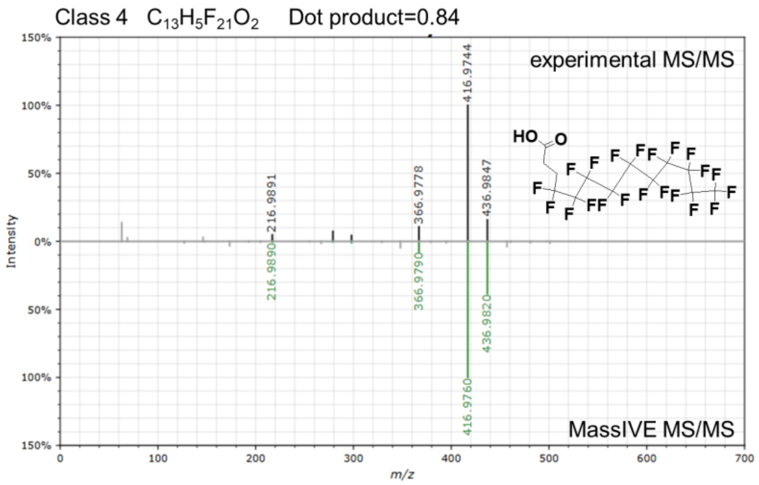


**Supplementary Figure 45. Mirror plot of 10:3 FTCA.**

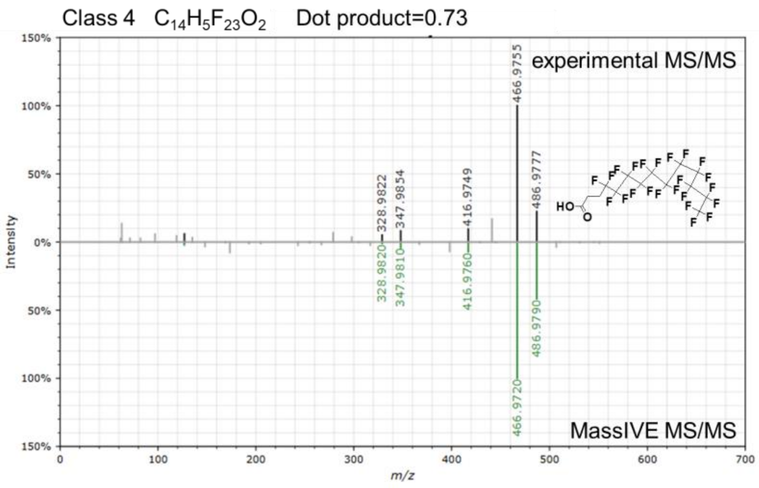


**Supplementary Figure 46. Mirror plot of 11:3 FTCA.**

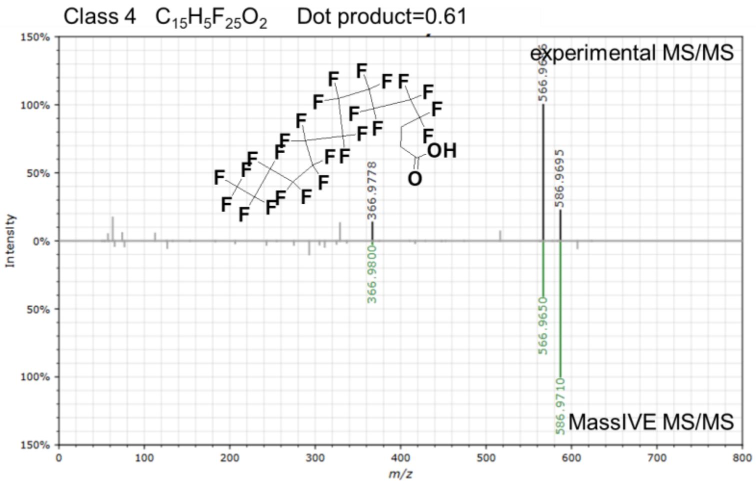


**Supplementary Figure 47. Mirror plot of 12:3 FTCA.**

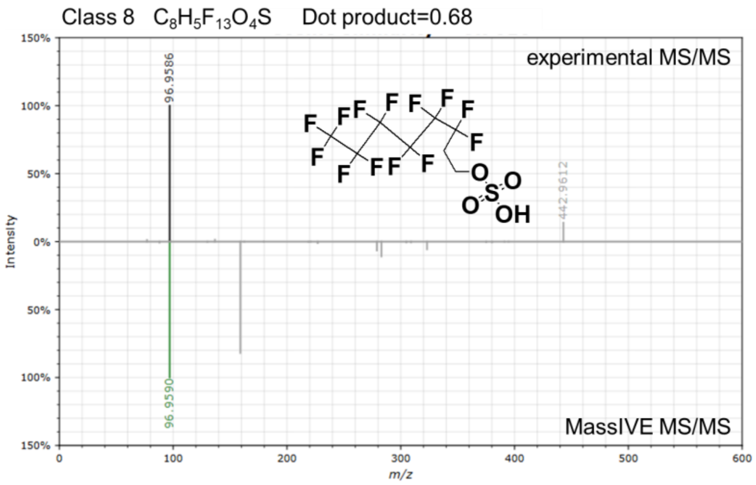


**Supplementary Figure 48. Mirror plot of 6:2 FTSF.**

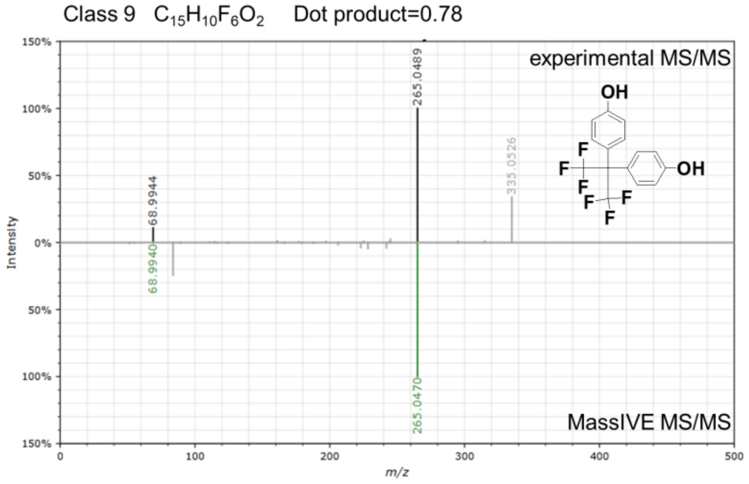


**Supplementary Figure 49. Mirror plot of BPAF.**

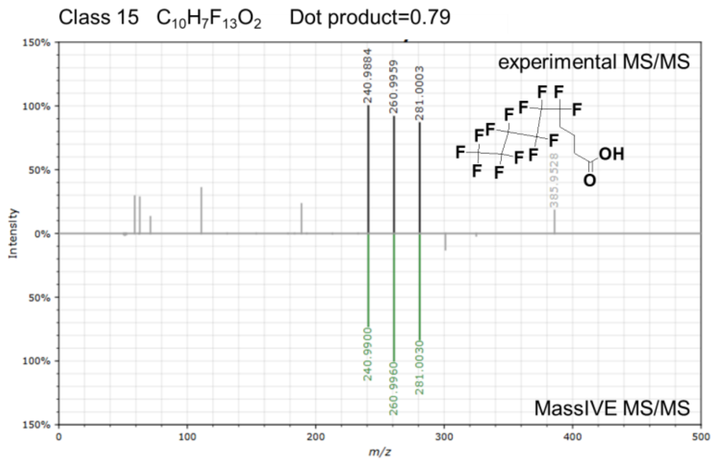


**Supplementary Figure 50. Mirror plot of 6:4 FTCA.**

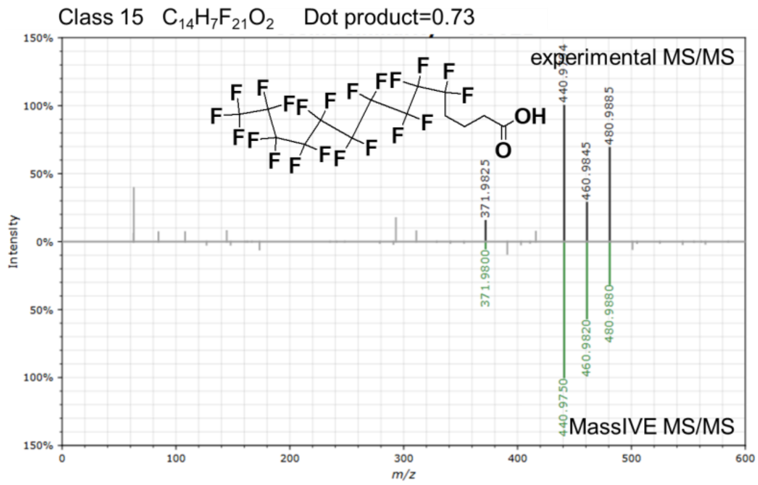


**Supplementary Figure 51. Mirror plot of 10:4 FTCA.**

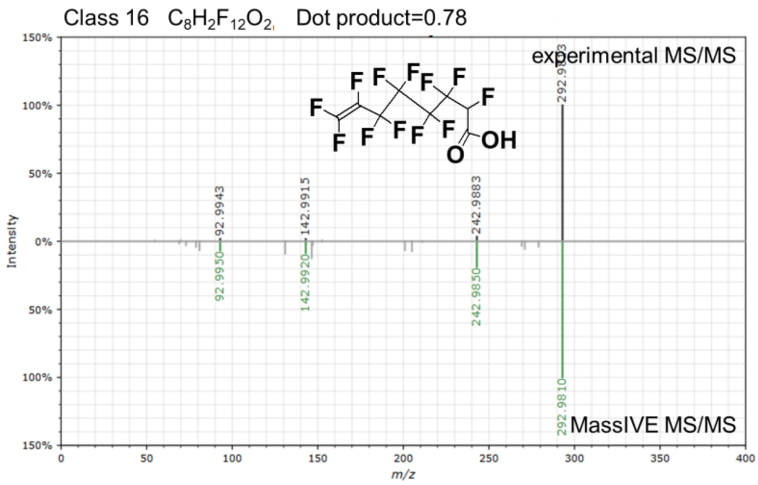


**Supplementary Figure 52. Mirror plot of dH-PFOA.**

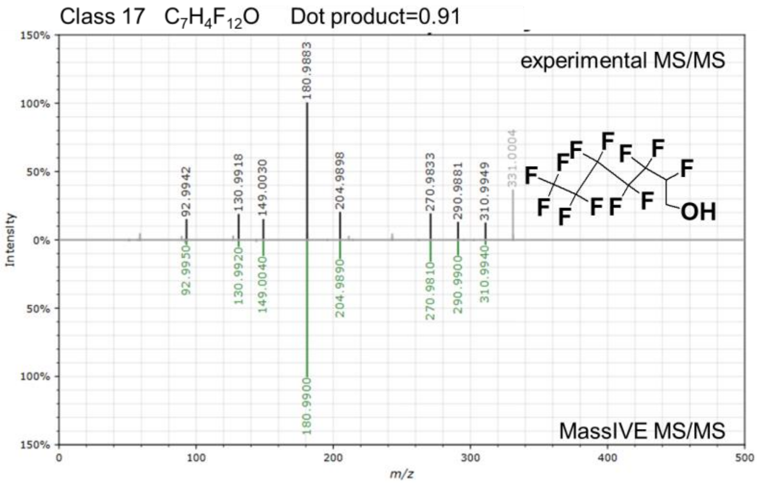


**Supplementary Figure 53. Mirror plot of H-6:1 FTOH.**

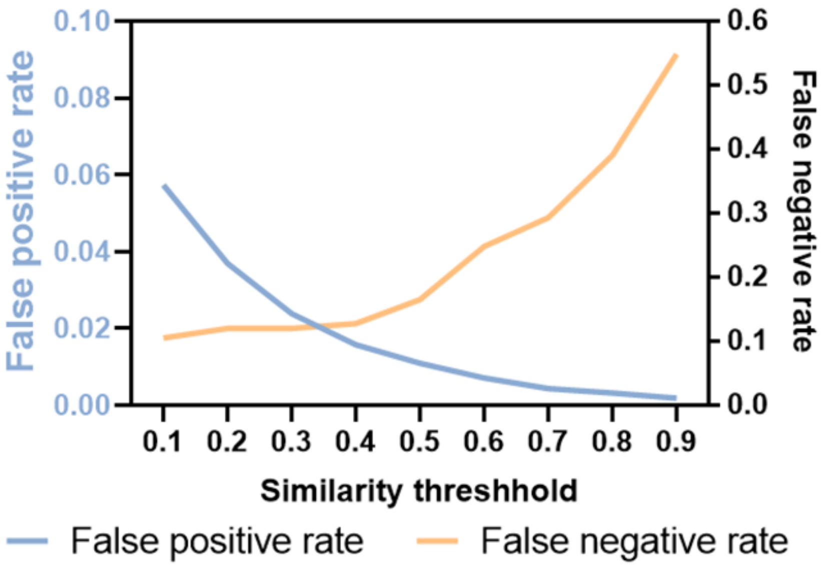


**Supplemetary Figure 54. The parameter selection of GNPS spectrum similarity using local spectra database.** The blue ones refer to false positive rate while the orange ones refer to the false negative rate. Source data are provided as a Source Data file.

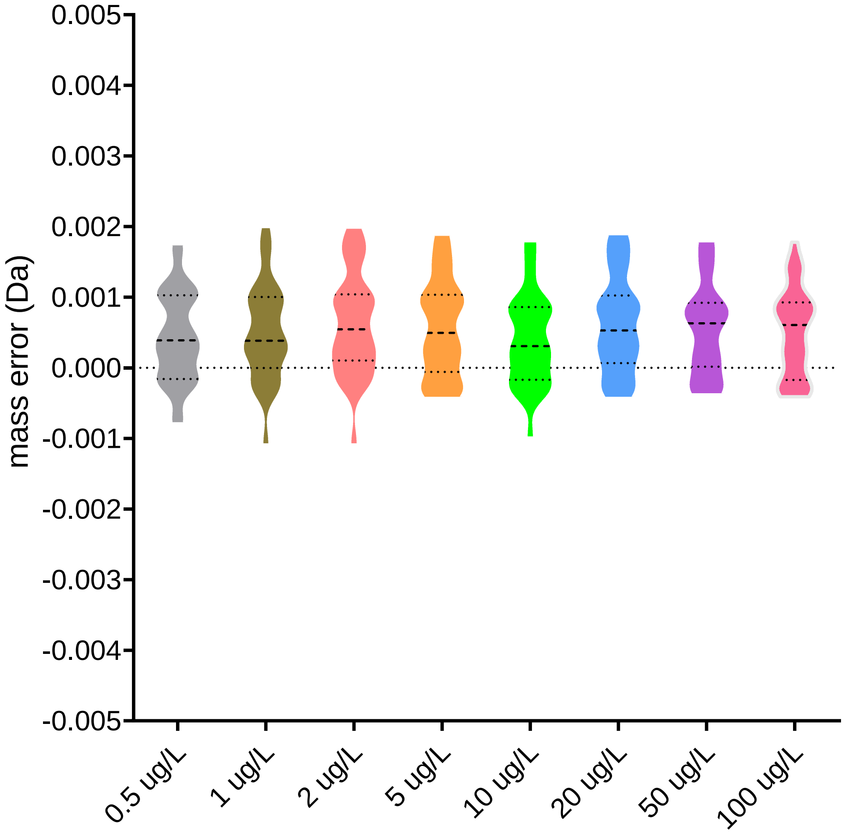


**Supplementary Figure 55. Precursor mass errors of 35 PFAS standards under concentration from 0.5-100 ug/L on our instrument.** Source data are provided as a Source Data file.

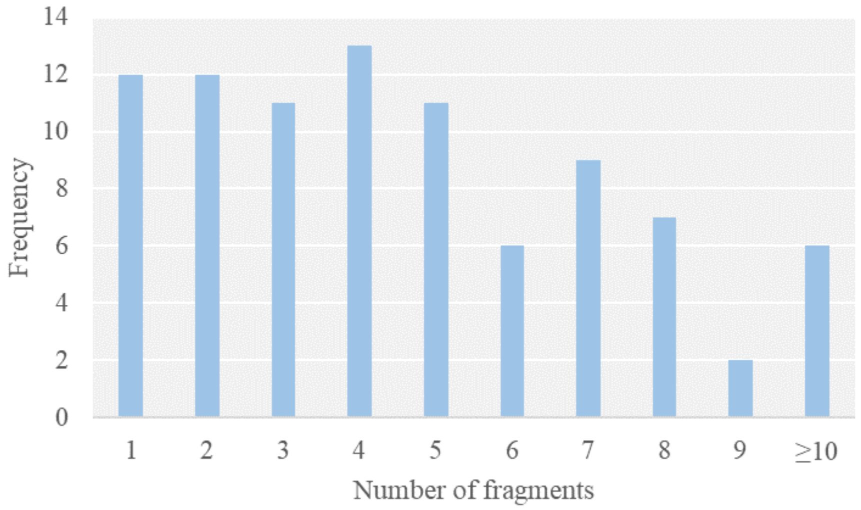


**Supplementary Figure 56. Numbers of effective fragments of 94 identified PFAS.** Source data are provided as a Source Data file.

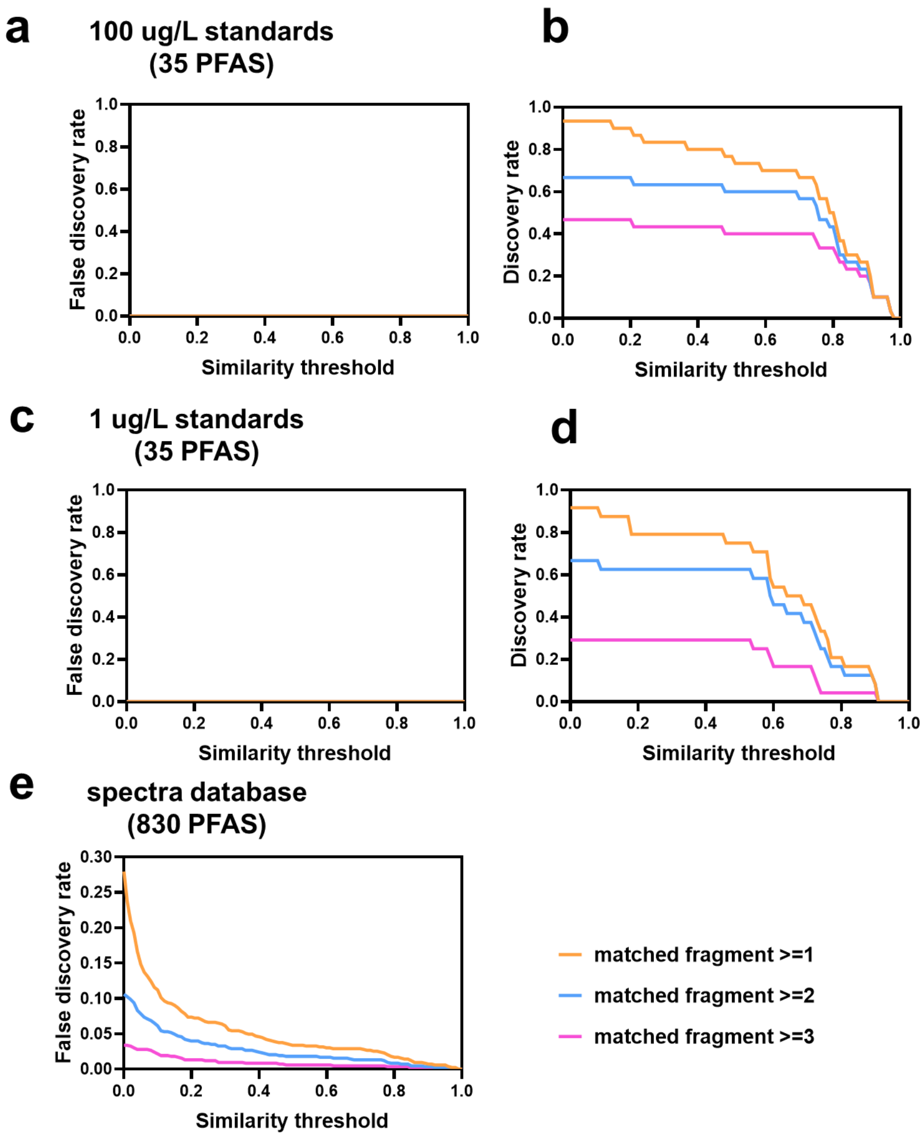


**Supplementary Figure 57. MASST search parameters optimization. a** the false discovery rate and **b** discovery rate of 35 PFAS from 100 ug/L standards, 35 PFAS were search against 830 PFAS, 16734 local non-PFAS spectra and 2207 blank sample spectra. **c** the false discovery rate and **d** discovery rate of 35 PFAS from 1 ug/L standards. **e** the false discovery rate of 830 PFAS, 830 PFAS were search against 16734 local non-PFAS spectra and 2207 blank sample spectra. Source data are provided as a Source Data file.
