# Supplementary material for: Two-layer homolog network approach for PFAS nontarget screening and retrospective data mining
Source: Nat Commun. 2025 Jan 15;16:688. doi: 10.1038/s41467-025-56035-1 (PMC11735632; doi:10.1038/s41467-025-56035-1)
Supplement: Supplementary file 2 — Reporting Summary [file 41467_2025_56035_MOESM2_ESM.pdf]

nature portfolio

Corresponding author(s): Si Wei

Last updated by author(s): Dec 20, 2024

Reporting Summary

Nature Portfolio wishes to improve the reproducibility of the work that we publish. This form provides structure for consistency and transparency in reporting. For further information on Nature Portfolio policies, see our Editorial Policies and the Editorial Policy Checklist.

Statistics

For all statistical analyses, confirm that the following items are present in the figure legend, table legend, main text, or Methods section.

n/a	Confirmed	
☐	☒	The exact sample size (*n*) for each experimental group/condition, given as a discrete number and unit of measurement
☐	☒	A statement on whether measurements were taken from distinct samples or whether the same sample was measured repeatedly
☐	☒	The statistical test(s) used AND whether they are one- or two-sided<br>*Only common tests should be described solely by name; describe more complex techniques in the Methods section.*
☐	☒	A description of all covariates tested
☐	☒	A description of any assumptions or corrections, such as tests of normality and adjustment for multiple comparisons
☐	☒	A full description of the statistical parameters including central tendency (e.g. means) or other basic estimates (e.g. regression coefficient) AND variation (e.g. standard deviation) or associated estimates of uncertainty (e.g. confidence intervals)
☐	☒	For null hypothesis testing, the test statistic (e.g. F, t, r) with confidence intervals, effect sizes, degrees of freedom and P value noted<br>*Give P values as exact values whenever suitable.*
☐	☒	For Bayesian analysis, information on the choice of priors and Markov chain Monte Carlo settings
☐	☒	For hierarchical and complex designs, identification of the appropriate level for tests and full reporting of outcomes
☐	☒	Estimates of effect sizes (e.g. Cohen's d, Pearson's r), indicating how they were calculated

Our web collection on statistics for biologists contains articles on many of the points above.

Software and code

Policy information about availability of computer code

Data collection	No software is used in this process.
Data analysis	MS-DIAL ver 4.70 used for peak picking. FluoroMatch 4.5 and FindPFΔS 0.1.0 used for comparing, Gephi0.10.1 used for networking and community detection. R 3.6 and python 3.8 used for drawing plots.<br>The code used in this study are available in Zendo (https://zenodo.org/records/13482320) The code used in this study are available in Zendo (https://zenodo.org/records/13482320).

For manuscripts utilizing custom algorithms or software that are central to the research but not yet described in published literature, software must be made available to editors and reviewers. We strongly encourage code deposition in a community repository (e.g. GitHub). See the Nature Portfolio guidelines for submitting code & software for further information.

Data

Policy information about availability of data

All manuscripts must include a data availability statement. This statement should provide the following information, where applicable:

- Accession codes, unique identifiers, or web links for publicly available datasets
- A description of any restrictions on data availability
- For clinical datasets or third party data, please ensure that the statement adheres to our policy

The raw mass spectrometry files of the samples and the local database are available on MASSIVE with the accession number MSV000095719 (doi:10.25345/

C5251FX35).

Research involving human participants, their data, or biological material

Policy information about studies with human participants or human data. See also policy information about sex, gender (identity/presentation), and sexual orientation and race, ethnicity and racism.

Reporting on sex and gender	There is no related data.
Reporting on race, ethnicity, or other socially relevant groupings	There is no related data.
Population characteristics	There is no related data.
Recruitment	There is no related data.
Ethics oversight	There is no related data.

Note that full information on the approval of the study protocol must also be provided in the manuscript.

Field-specific reporting

Please select the one below that is the best fit for your research. If you are not sure, read the appropriate sections before making your selection.

☐ Life sciences ☐ Behavioural & social sciences ☒ Ecological, evolutionary & environmental sciences

For a reference copy of the document with all sections, see nature.com/documents/nr-reporting-summary-flat.pdf

Ecological, evolutionary & environmental sciences study design

All studies must disclose on these points even when the disclosure is negative.

Study description	We developed a two-layer homolog network approach for per- and polyfluoroalkyl substances(PFAS) nontarget screening using mass spectrometry.
Research sample	Waterproof products and related industrial sludge samples.
Sampling strategy	These product samples were purchased in the market. The sludge samples were collected from the drainage of fluorochemical industry. A few samples were collected since this study focused on the discovery of PFAS. The numbers of samples could support the identify of emerging and novel PFAS
Data collection	No data related.
Timing and spatial scale	Data are collected in Osaka, Japan in January 2022.
Data exclusions	No data is excluded from any analysis.
Reproducibility	Our findings were based on high-resolution mass spectrometer, detailed parameters are given.
Randomization	No data related.
Blinding	No data related.

Did the study involve field work? ☐ Yes ☒ No

Reporting for specific materials, systems and methods

We require information from authors about some types of materials, experimental systems and methods used in many studies. Here, indicate whether each material, system or method listed is relevant to your study. If you are not sure if a list item applies to your research, read the appropriate section before selecting a response.

Materials & experimental systems

n/a	Involved in the study
☒	☐ Antibodies
☒	☐ Eukaryotic cell lines
☒	☐ Palaeontology and archaeology
☒	☐ Animals and other organisms
☒	☐ Clinical data
☒	☐ Dual use research of concern
☒	☐ Plants

Methods

n/a	Involved in the study
☒	☐ ChIP-seq
☒	☐ Flow cytometry
☒	☐ MRI-based neuroimaging

Plants

Seed stocks	No related data.
Novel plant genotypes	No related data.
Authentication	No related data.
