# Supplementary material for: Two-layer homolog network approach for PFAS nontarget screening and retrospective data mining
Source: Nat Commun. 2025 Jan 15;16:688. doi: 10.1038/s41467-025-56035-1 (PMC11735632; doi:10.1038/s41467-025-56035-1)
Supplement: Supplementary file 3 — Description of Additional Supplementary Files [file 41467_2025_56035_MOESM3_ESM.pdf]

Supplementary Data 1:
The class, name, formula, precursor m/z, RT, and confidence level of identified PFAS

Supplementary Data 2:
The list of emerging PFAS compiled from previous literatures and reviews

Supplementary Data 3:
Links to MASST Work for Matched PFAS

Supplementary Data 4:
The abbreviations, supplier, and purity of PFAS standards.
